# Spectroscopic Identification of the Charge Transfer State in Thiophene/Fullerene Heterojunctions: Electroabsorption Spectroscopy from GW/BSE Calculations

**DOI:** 10.1021/acs.jpcc.3c03734

**Published:** 2023-08-04

**Authors:** Smruti Ranjan Sahoo, Charles H. Patterson

**Affiliations:** School of Physics, Trinity College Dublin, Dublin 2, D02 PN40, Ireland

## Abstract

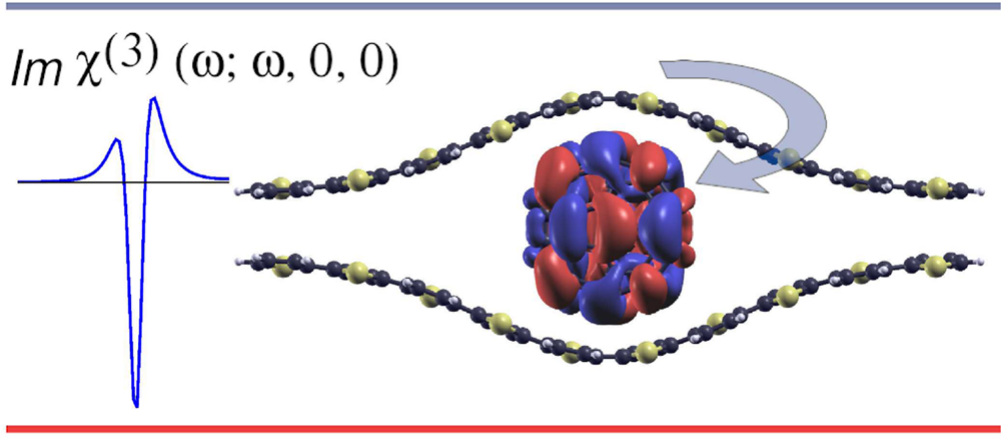

Creation of charge transfer (CT) states in bulk heterojunction systems such as C_60_/polymer blends is an important intermediate step in the creation of carriers in organic photovoltaic systems. CT states generally have small oscillator strengths in linear optical absorption spectroscopy owing to limited spatial overlap of electron and hole wave functions in the CT excited state. Electroabsorption spectroscopy (EA) exploits changes in wave function character of CT states in response to static electric fields to enhance detection of CT states via nonlinear optical absorption spectroscopies. A 4 × 4 model Hamiltonian is used to derive splittings of even and odd Frenkel (FR) excited states and changes in wave function character of CT excited states in an external electric field. These are used to explain why FR and CT states yield EA lineshapes which are first and second derivatives of the linear optical absorption spectrum. The model is applied to ammonia–borane molecules and pairs of molecules with large and small B–N separations and CT or FR excited states. EA spectra are obtained from differences in linear optical absorption spectra in the presence or absence of a static electric field and from perturbative sum over states (SOS) configuration interaction singles χ^(2)^ and χ^(3)^ nonlinear susceptibility calculations. Good agreement is found between finite field (FF) and SOS methods at field strengths similar to those used in EA experiments. EA spectra of three C_60_/oligothiophene complexes are calculated using the SOS method combined with GW/BSE methods. For these C_60_/oligothiophene complexes, we find several CT states in a narrow energy range in which charge transfer from the thiophene HOMO level to several closely spaced C_60_ acceptor levels yields an EA signal around 10% of the signal from oligothiophene.

## I Introduction

Charge transfer (CT) states, in which the electron and hole in a bound exciton are localized on distinct molecular entities, are important intermediate states in creation of carriers in organic photovoltaic systems.^1−5^ They are also important in biological systems such as PS II.^6−8^ They may be formed following photoexcitation by a photosensitizer and relaxation to a CT state, or they may be created directly by photoabsorption, with a very low absorption cross section. Spectroscopic identification of CT states at sub-band gap energies remains difficult because of the low cross section^9^ and because there are other weak absorptions from excitations such as polarons,^10,11^ vibrational replicas of the fundamental band, etc. in the same energy range. Electroabsorption (EA) spectroscopy^12,13^ offers a way of enhancing the optical cross section of CT states relative to local or Frenkel (FR) excitations by exploiting the large Stark shift of their excited state dipole moments in a static external electric field. In EA spectroscopy, changes in the optical absorption spectrum of a thin film induced by a low frequency applied electric field are measured. EA can be modeled using the Liptay equation,^14,15^ which is a classical approach based on molecular polarizability derivatives with respect to external field frequency, or using a quantum mechanical sum over states (SOS) nonlinear optical susceptibility.^16,17^ The connection between these approaches has been discussed by Saito and co-workers.^18^

Here we present a model for EA based on a pair of two level systems with inversion symmetry, perform finite field (FF) and perturbative sum over states (SOS) calculations for a model ammonia-borane system to validate the SOS method, and then apply it to EA spectra of oligothiophene-C_60_ complexes. The model is based on a 4 × 4 Hamiltonian with two FR and two CT excited states.^19^ EA spectra are commonly fitted to first and second derivative lineshapes of the linear optical absorption spectrum.^20^ Splittings of FR states induced by the Coulomb interaction in excited singlet states and strong changes in wave function character of CT states induced by an external field are used to explain how first and second derivative lineshapes arise in EA spectra of model systems constructed from ammonia and borane molecules. Δ*A*(ω) is the change in linear optical absorption spectrum induced by an external electric field. Calculations of Δ*A*(ω) are performed for these model systems using FF configuration interaction singles (CIS) calculations and by the SOS perturbation theory method. Results from the two approaches are in excellent agreement, validating the use of the SOS approach combined with GW/BSE calculations to calculate EA spectra for C_60_/oligothiophene systems.

From a phenomenological viewpoint, changes in polarization induced by a static external electric field may occur through changes in the polarizability or via a Stark shift of the excitation energy when there is a difference in the dipole moments of the excited and ground states. The former and latter are generally regarded as the mechanisms by which FR and CT states are observed in EA spectroscopy.^14,21^ In noncentrosymmetric molecular crystals, an EA signal which is linear in the static electric field may be observed via the linear electrooptic effect.^16,17,22^ This is governed by a second order χ^(2)^ nonlinear susceptibility. However, in centrosymmetric or disordered systems, symmetry or averaging over random molecular orientations means that the EA signal is governed by a third order, χ^(3)^ susceptibility,^16−18,21^ which is quadratic in the static electric field and the linear part vanishes.

Polythiophene–fullerene systems are the archetypal donor–acceptor bulk heterojunction (BHJ) organic photovoltaics.^23,24^ Reviews by Piliego and Loi,^1^ Gao and Inganäs,^2^ and Vandewal^3^ cite direct observation of CT state absorption and emission by C_60_ polymer blends. Using electroluminescence (EL) measurements, Tvingstedt and co-workers^25^ reported EL from poly(3-hexylthiophene-2,5-diyl)/phenyl-C61-butyric acid methyl ester (P3HT/PCBM) blends at 1.0 eV and in the range 1.3 to 1.4 eV in a poly(*p*-phenylenevinylene) (PPV)/PCBM blend. Similarly, Faist and co-workers observed emission at 0.89 eV from a P3HT/PCBM blend and in the range 0.97 to 1.32 eV in similar polymer/fullerene blends.^9^ Veldman and co-workers^26^ estimated the CT energy in P3HT/PCBM to be 0.99 eV and a range of CT energies in related systems to lie in the range 1.11 to 2.13 eV. A strong correlation between the energy of the CT state and the open circuit voltage of an organic PV cell made from a particular blend has also been noted.^26^ Optical signals from sub-band gap states in polymer–fullerene BHJ systems have been reported at 0.9 eV by photothermal deflection spectroscopy (PDS) in P3HT/PCBM.^10^ Sanden and co-workers reported peaks in P3HT/PCBM samples at 0.33, 1.26, and 1.8 eV^27^ in photoinduced absorption (PIA) measurements. Signals attributed to CT states in these systems have been observed in EA spectroscopy.^20,23,28,29^ However, polaron absorption in conjugated polymers also lies in the range 1.0 to 1.6 eV, where CT states also lie. Polaron optical absorption in a conjugated polymer has recently been reinterpreted.^11^

CT states in oligothiophene/C_60_ and similar systems have been modeled using time-dependent density functional theory (TDDFT) applied to single donor–acceptor pairs.^10,30−33^ We reported EA calculations on tetrathiophene dimers^19^ and found that the main features arise from the main 1B_1*u*_ thiophene FR transition and the CT state; the former exhibits a first derivative EA line shape and the latter a second derivative shape. Both are observed in experiment on polycrystalline P3HT.^34^ Electronic transitions probed by optical absorption in C_60_ in solution^35−37^ and in the gas phase^38^ have been reported, and optical excitations and vibronic interactions in C_60_ have been analyzed by Negri and co-workers.^39−41^ EA spectra from C_60_ thin films have been reported a number of times^42−45^ and model Hamiltonian calculations have been used to analyze these spectra.^46^ Calculations of two photon absorption spectra of C_60_ have also been reported.^47,48^

The organization of the remainder of this paper is as follows: the SOS and phenomenological models for EA spectroscopy are outlined and illustrated using the BH_3_:NH_3_ adduct and details of computational parameters and methods are given in Section II; results of EA calculations on the BH_3_:NH_3_ adduct and its dimer from FF and SOS approaches are compared; the linear and nonlinear optical absorption by gas phase C_60_ and EA from oligothiophene/C_60_ systems are presented in Section III; opportunities presented by EA spectroscopy for identification of CT states in BHJ polymer-C_60_ systems are considered in Section IV and a summary of results and conclusions are presented in Section IV.B.

## II Theory and Computational Methods

### II.A Electroabsorption Spectroscopy

EA is a spectroscopy in which changes in the linear optical absorption spectrum, Δ*A*, of a thin film induced by a static (or, in practice, low frequency) external electric field, **F**, are measured. It can be modeled using the Liptay equation,^14,15^
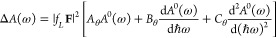
1where *f*_*L*_ is a local field factor, **F** is the quasi-static electric field, and *A*^0^(ω) is the linear optical absorption when **F** = 0 and θ is the angle between the external static and optical frequency electric fields.

The Liptay equation is a classical approach based on first and second molecular polarizability derivatives with respect to optical field frequency, ω. EA can also be modeled using the imaginary part of a third order nonlinear susceptibilty, χ^(3)^(−ω; ω, 0, 0),^16^ with two static and one optical frequency input electric fields. The relationship between Δ*A*(ω) and χ^(3)^ is,^18^
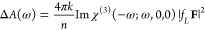
where *k* and *n* are the optical wavenumber and material refractive index, respectively.

Saito and co-workers have given a detailed analysis of the correspondence of these two approaches.^18^ The Liptay equation is quadratic in the static field. The absence of any linear dependence on this field arises because macroscopic averages over random molecular orientations in a macroscopic sample result in zero net signal at first order in the field.^15,18^ However, in a noncentrosymmetric crystal the leading order response of a sample in an external field is the linear electrooptic (or Pockels) effect.^17^ The linear electrooptic spectrum is obtained from the imaginary part of,
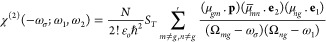
2The input optical field frequency, ω_1_ = ω, input quasi-static field frequency, ω_2_, and the resultant polarization frequency, ω_σ_, satisfy ω_σ_ = ω_1_ + ω_2_ ∼ ω, where *N* is the number density of molecules, *S*_*T*_ is a permutation operator which permutes pairs (−ω_σ_, **p**), (ω_1_, **e**_1_), (ω_2_, **e**_2_) in which **p**, **e**_1_, and **e**_2_ are unit vectors for the corresponding electric fields. Ω_*mg*_ is the transition frequency for the ground state |*g*⟩ to excited state |*m*⟩ minus a small imaginary lifetime factor, *i*Γ/2. **μ**_*gm*_ = ⟨*g*|**μ**|*m*⟩ is the transition dipole moment connecting those states. The overbar on the transition moment  indicates the difference in excited and ground state permanent dipole moments when *m* = *n*, i.e.,  = ⟨*m*|**μ**|*n*⟩ – ⟨*g*|**μ**|*g*⟩δ_*mn*_.

The third order optical susceptibility^16,17^ which describes the EA spectrum^15,18^ contains one-photon,
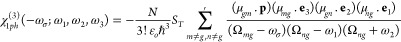
3and two-photon terms,
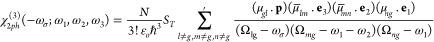
4Frequencies in eq 3 and 4 satisfy ω_σ_ = ω_1_ + ω_2_ + ω_3_ ∼ ω, The EA spectrum is obtained from the imaginary part of χ^(3)^(−ω; ω_1_, ω_2_, ω_3_), where ω_1_ = ω is the incident light frequency and ω_2_ = ω_3_ ∼ 0, is the quasi-static external field frequency, which has a field strength around 10^7^ V m^–1^ in experiment. Here *S*_*T*_ generates all pairwise permutations of (−ω_σ_, **p**), (ω_1_, **e**_1_), (ω_2_ = 0, **e**_2_), and (ω_3_ = 0, **e**_3_) (see ref (17), eq 4.120).

### II.B Hamiltonian and Spectral Lineshapes

**Figure 1:**
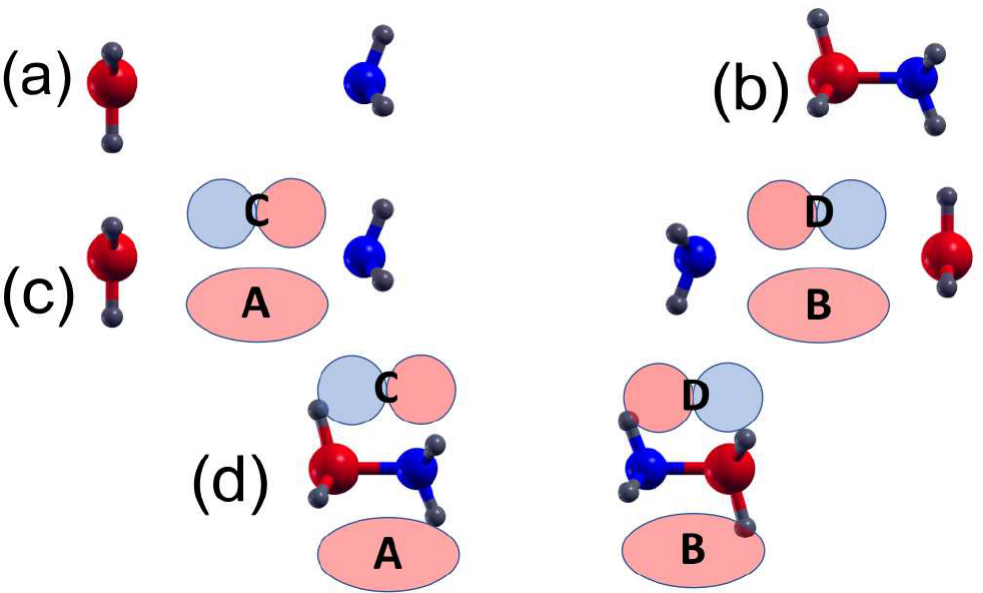
Molecular configurations of adducts and separated BH_3_ and NH_3_ molecules. (a) BH_3_ and NH_3_ molecules, *r*(BN) 5.0 Å, (b) BH_3_:NH_3_ adduct monomer, *r*(BN) = 1.6 Å; (c) BH_3_ and NH_3_ molecule dimer, *r*(BN) = 5.0 Å, *r*(NN) = 6.6 Å; (d) BH_3_:NH_3_ adduct dimer, *r*(BN) = 1.6 Å, *r*(NN) = 4.6 Å. Parts c and d also show local orbitals A–D which make up four frontier orbitals in dimers. The phase of orbital D is chosen for consistency with the model in ref (19).

In a recent publication^19^ we described the simplest model Hamiltonian which contains both FR and CT excitations in symmetric pairs of cofacial tetrathiophene molecules. Here we apply the same model to an ammonia–borane system to illustrate the connection between Δ*A*(ω) spectral lineshapes and the FR or CT character of excited states. We consider the Δ*A*(ω) spectrum of a pair of BH_3_ and NH_3_ molecules that share a common molecular axis, denoted *z* (Figure 1a,b). At short BN distances, these molecules form a bound adduct, which is a small molecule without CT transitions (Figure 1b). At larger BN distances (Figure 1a), however, the molecules have several CT transitions, because of their donor–acceptor character. This system lacks inversion symmetry, and its leading nonlinear response is described by a χ^(2)^ susceptibility. In contrast, the leading nonlinear response of the centrosymmetric systems containing dimers shown in Figure 1c,d is χ^(3)^.

For the model Hamiltonian applied to the ammonia–borane system, we assume that each NH_3_–BH_3_ pair has occupied σ states composed of local orbitals *A* and *B* and unoccupied σ* states composed of orbitals *C* and *D*. Linear combinations of these form the *H* – 0 (HOMO), *H* – 1, *L* + 0 (LUMO) and *L* + 1 orbitals (Figure 1). Dimer *H* – 1 and *H* – 0 orbitals are  and  and dimer *L* + 0 and *L* + 1 orbitals are  and . For simplicity, it is assumed that intermolecular distances are large enough that overlaps ⟨*A*|*B*⟩ and ⟨*C*|*D*⟩ are negligible. We previously considered a minimal Hamiltonian for triplet states^19^ in which a CIS Hamiltonian matrix element is,
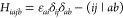
5ε_*ai*_ is the HOMO–LUMO gap (assumed the same for *H* – 0 → *L* + 0 and *H* – 1 → *L* + 0, etc.). Chemist’s notation is used for two-electron integrals (*ij*|*kl*), in which orbitals *i* and *j* share the same electron coordinate and *k* and *l* share the other electron coordinate. Diagonal and off-diagonal (*ij*|*ab*) two-electron matrix elements have the following approximate forms,
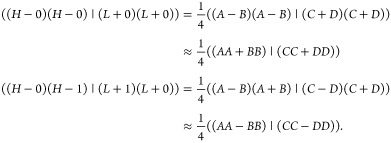
(*AA*|*BB*) etc. are Coulomb integrals over local orbitals *A*, *B*, etc. Transitions in the Hamiltonian are ordered as *H* – 1 → *L* + 0, *H* – 1 → *L* + 1, *H* – 0 → *L* + 0, and *H* – 0 → *L* + 1. Each diagonal element has the same (*ij*|*ab*) two electron part and each nonzero off-diagonal part is the same when intermolecular overlaps ⟨*A*|*B*⟩ and ⟨*C*|*D*⟩ are neglected. The odd/even symmetry of the molecular wave functions with respect to the center of inversion means that only four off-diagonal terms in the Hamiltonian are nonzero.^19^

The Hamiltonian in eq 5 accounts for the Coulombic behavior of FR and CT state energies as a function of the dimer separation for *triplet* states. The Hamiltonian for singlet states in this model has the term 2(*ia*|*jb*), in addition to terms in eq 5. The degenerate triplet FR state energy is ε_*ai*_–((*AA*|*CC*)+(*BB*|*DD*))/2 and the degenerate triplet CT state energy is ε_*ai*_–((*AA*|*DD*)+(*BB*|*CC*))/2. The corresponding wave functions for the first eigenvalue^19^ are,
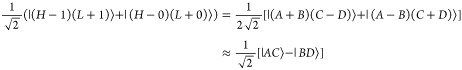
6
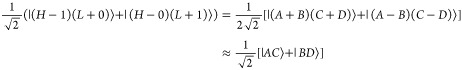
7These states are linear combinations in which the excited electron and hole reside on the same molecule in each configuration and combine with different phases. These Frenkel states are denoted as FR1 and FR2. The corresponding wave functions for the eigenvalue ε_*ai*_–((*AA*|*DD*)+(*BB*|*CC*))/2^19^ are,
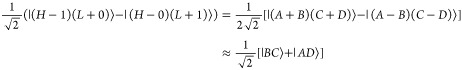
8
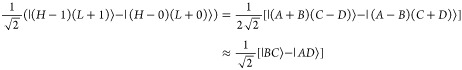
9These states are in- and out-of-phase combinations in which the excited electron and hole reside on either molecule, i.e. CT1 and CT2 charge transfer states. The additional terms in the model Hamiltonian which arise from the 2(*ia*|*jb*) term for singlet states are,
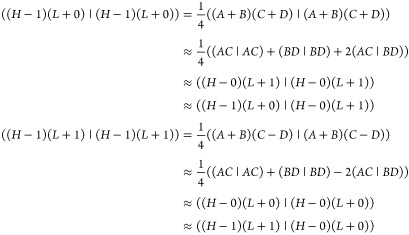
Approximate equalities here apply when overlaps ⟨*A*|*B*⟩ and ⟨*C*|*D*⟩ are neglected. The 2(*ia*|*jb*) term in the singlet state Hamiltonian increases the FR2 state energy by 2(*AC*|*BD*) and lowers the FR1 state by the same amount. This is the dipole–dipole coupling of the monomer transition moments. Energies of the CT states are unchanged by the 2(*ia*|*jb*) term in the Hamiltonian equation when overlaps are neglected.

In an external field, **F**, the interaction Hamiltonian with a molecular permanent or transition dipole moment, **μ**_*ij*_, is,
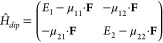
10*E*_1_ and *E*_2_ are the energies of pairs of states (FR1 and FR2 or CT1 and CT2) in the absence of the field and **μ**_12_ is the dipole transition moment connecting them. Nonzero matrix elements of – **μ**_*ij*_.**F** are,
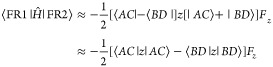
and
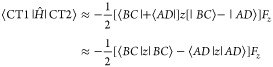
Changes in character of FR and CT states induced by the Coulomb interaction and an external field differ. FR states are split by the Coulomb interaction but are mixed to a limited extent by an external field if |*E*_2_ – *E*_1_| > |**μ**_12_·**F**|. In contrast, CT states are not split by the Coulomb interaction but couple strongly to an external field. These conclusions are based on the assumption of neglect of overlap of wave functions on separate molecules and pure FR or CT character for the states. Therefore, for a state with strong FR character, large splittings induced by the Coulomb interaction and small splittings induced by an external field are expected, while for states with strong CT character, small splittings induced by the Coulomb interaction are expected. Large changes in wave function character are also expected for CT states because of their small energetic splitting: an external field is expected to induce strong mixing of the CT1 and CT2 states and thereby induce excited states with large permanent dipole moments.

**Figure 2:**
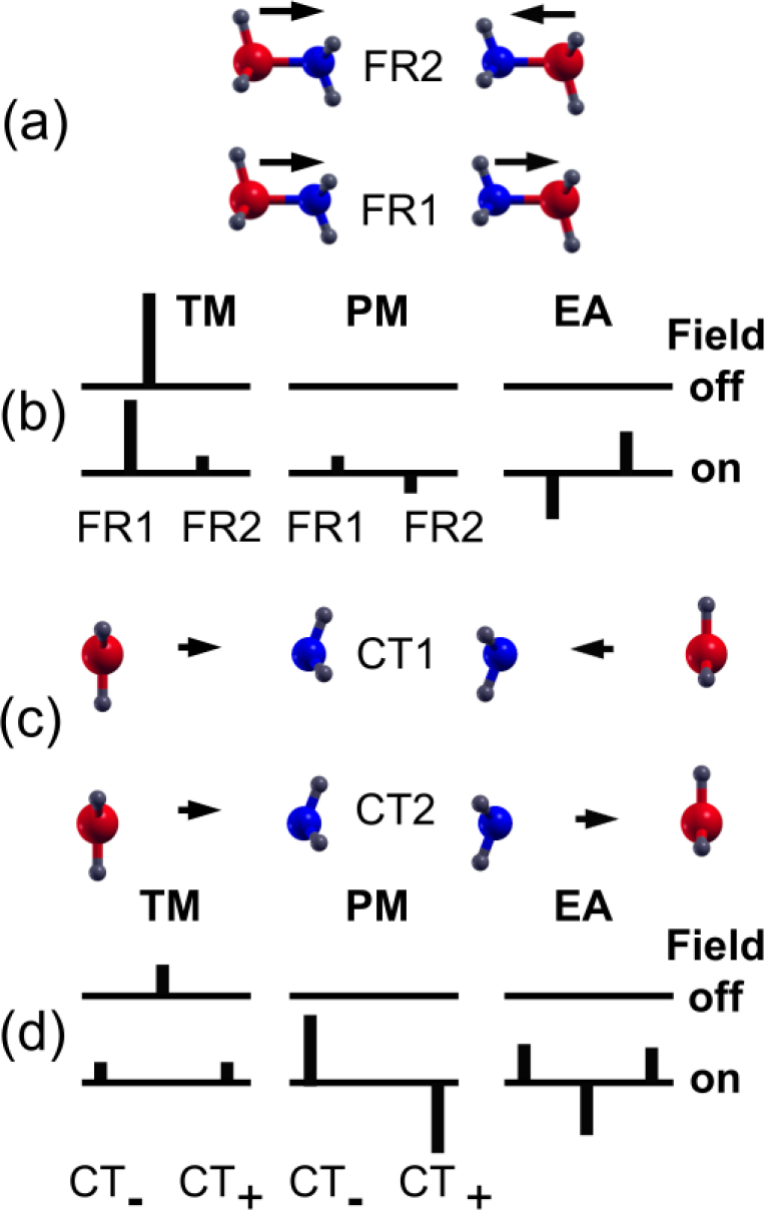
EA spectra of FR and CT excitations. (a) Relative phases of transition dipole moments (TM) in BH_3_:NH_3_ pairs in FR modes, FR1 and FR2, indicated by arrows. (b) TM of modes FR1 and FR2 with an external electric field on and off, excited state permanent dipole moments (PM) with an external electric field on and off, and the EA spectrum. (c) Relative phases of TM in separated BH_3_ NH_3_ pairs in CT modes, CT1 and CT2, indicated by arrows. (d) TM of modes CT1 and CT2 with an external electric field on and off, excited state PM with an external electric field on and off, and the EA spectrum. The TM in FR modes is larger than the TM in CT modes as indicated by arrow length and the PM in CT modes is larger than in FR modes resulting in a larger Stark shift in CT modes. Excited states acquire PM of opposite sign in external electric field.

The effect of these couplings on excitation energies and Δ*A*(ω) spectral lineshapes for FR and CT states is illustrated in Figure 2. FR1 and FR2 (Figure 2a) are excitations in which local FR excitations of each adduct combine in-phase (FR1) or out-of-phase (FR2). The relatively large splitting of FR1 and FR2 states means that |*E*_2_ – *E*_1_| > |**μ**_12_·**F**| at typical EA experimental field strengths and eigenvectors for states of mainly FR character are *not* strongly mixed by the external field. For states of CT character, there is full mixing of states. Permanent dipole moments of CT_+_(**F**) and CT_–_(**F**),
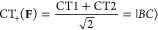
11and
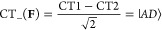
12correspond to full electron transfer from donor to acceptor, parallel or antiparallel to the applied field. Interactions of FR and CT states with an electric field (described qualitatively here) are revisited in Section III.

We now consider the implications of these two limits of interaction of pairs of excited states with an external electric field for the EA spectrum. Changes in the absorption spectrum of a pair of FR oscillators are illustrated in Figure 2b. In the absence of an external field, the FR1 state with in-phase intramolecular dipole oscillators has a nonzero transition moment (TM) while the FR2 state has zero transition moment. When an external field is applied, the FR1 and FR2 state eigenvectors mix to a small degree, resulting in small permanent moments (PM) for the new states in the field. The transition moment for the optically active state is reduced, and the optically inactive FR2 state becomes weakly active. The difference in optical absorption with and without the field consists of a first derivative EA line shape (Figure 2b). The splitting of the transition energies induced by the external field is small in this case.

The CT2 state (Figure 2c) has a small transition moment in the absence of an external electric field, and the CT1 state is optically inactive. When an external field is applied, complete mixing of the states results in large permanent moments of opposite sign and equal magnitude in the new states as well as a large energy splitting. Changes in the absorption spectrum of a pair of CT oscillators are illustrated in Figure 2d. The EA spectrum derives from the original CT2 oscillator and two new oscillators with a large energy splitting, giving the familiar second derivative line shape associated with CT states.

Molecular configurations used in this section were chosen with and without inversion symmetry in order to create systems with the first nonlinear response at the second or third order in applied fields. In an amorphous BHJ structure inversion symmetry is lacking, however, the χ^(2)^ response is *linear* in the static external field and so averages to zero when a random ensemble of molecular orientations is chosen. Saito and co-workers have presented orientational averages for the *A*_θ_, *B*_θ_, and *C*_θ_ coefficients^18^ in eq 1. For this work the orientational average over the *C*_θ_ coefficient, ⟨*C*_θ_⟩, is the most relevant,
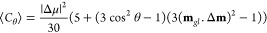
13Here |Δ**μ**| is the change in permanent dipole moment in an excited state, *l*, relative to the ground state, *g*, **m**_*gl*_ is a unit vector parallel to the **μ**_*gl*_ transition dipole moment, and Δ**m** is a unit vector parallel to the excited state permanent dipole moment. In the case where there is additional dipole coupling between excited state, *l*, and closely spaced excited state, *m*, additional terms which replace |Δ**μ**|^2^ by |**μ**_*lm*_|^2^ and Δ**m** by **m**_*lm*_ must be added.^18^ For the case where θ = 0 (χ_*xxxx*_^(3)^ or χ_*yyyy*_^(3)^) the numerical factor in eq 13 is 3/10 and for the case where θ = π/2 (χ_*xxyy*_^(3)^ or χ_*yyxx*_^(3)^) the numerical factor is 1/5.

### II.C Computational Details

#### II.C.1 Basis Sets and Codes

The Exciton code and methods used for GW/BSE calculations in this work have been described elsewhere.^49,50^ Calculations on ammonia-borane systems were performed using 6-311G basis sets for B, H and N^51^ and the CIS method in Exciton. Molecular geometry optimizations for thiophene and C_60_ systems were performed with the B3LYP functional and def2-TZVP basis sets^52^ using the Gaussian16 code.^53^ Geometry optimizations with C_60_ and oligothiophenes included the D3BJ dispersion correction in the DFT exchange-correlation functional.^54^ TDDFT calculations in this work used the Gaussian16 code, the ωB97X-D functional^55,56^ and def2-TZVP basis sets. def2-TZVP-RIFIT auxiliary basis sets^57^ were used for density fitting in GW/BSE calculations in the Tamm–Dancoff approximation (TDA). GW/BSE calculations on C_60_ were performed using the aug-cc-pVDZ basis set for C.^58,59^ Calculations on thiophene-C_60_ complexes were performed using smaller, modified def2-TZVP (C) and def2-SVP (H) and 6-311G* (S) basis sets with some high angular momentum functions omitted (Tables S1 to S3) to allow calculations to be performed on large systems. Calculations on C_60_ were repeated with the modified def2-TZVP basis and excitation frequencies, and linear and nonlinear optical absorption spectra reproduce well with the smaller basis set (Figure S1). GW/BSE calculations used the following molecular orbital active space ranges: C_60_ (61 to 697); T6-C_60_ (175 to 643); T6-C_60_-T6 (206 to 854); and T10-C_60_-T10 (423 to 1102). The first 400 excited states were used in EA spectra calculations. Corresponding cutoff energies for the 400th excited state in the three complexes used were 5.7, 6.0, and 6.9 eV.

#### II.C.2 Finite Field EA Calculations

External electric fields in FF calculations were generated by placing a charge of 4*e* or 16*e* 140 Å from the coordinate origin on the molecular axis, which yielded electric field strengths of 2.9 × 10^7^ V m^–1^ or 1.2 × 10^8^ V m^–1^ at the origin. The former is comparable to field strengths used in EA experiments. The latter is used to demonstrate changes in line shape with field strength, but is probably beyond the range encountered in experiments. The optical field is directed along the molecular axis. The static electric field results in splittings of CT state energies of order 50 meV; a line width of 40 meV was used in calculating optical absorption spectra. EA spectra were obtained by calculating the optical absorption spectrum using a CIS method in the presence and absence of this field and subtracting one from the other.

#### II.C.3 Screening in GW@HF/BSE Calculations

**Figure 3:**
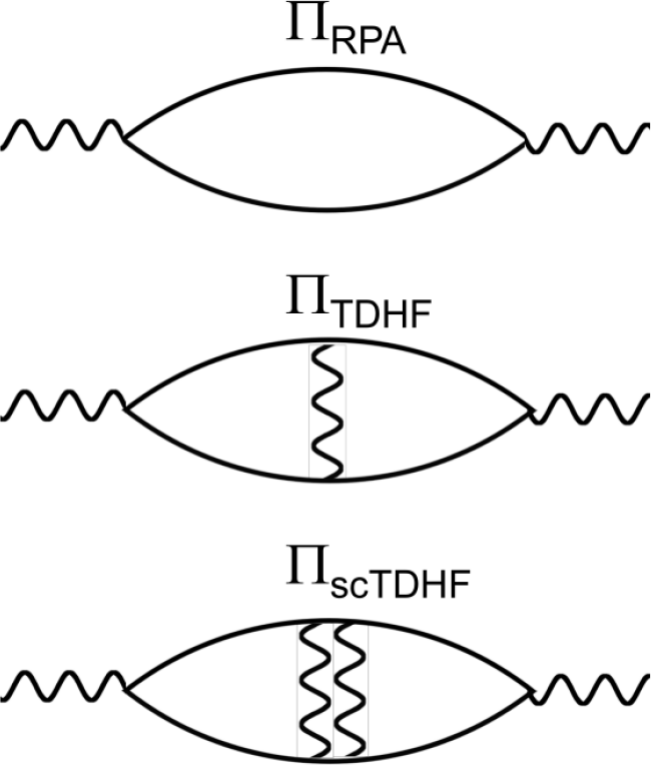
RPA, TDHF, and scTDHF polarizabilities in the screened interactions used in this work.

Optical absorption spectra of C_60_ from GW@HF/BSE calculations presented in Section III were obtained using SCF HF wave functions and single-particle eigenvalues as starting points for the GW self-energy and BSE Hamiltonian. More commonly, GW calculations are performed by using SCF DFT wave functions and single-particle eigenvalues as starting points. Screening of the electron–hole interaction is a third important input to a GW/BSE calculation. Typically, screening of HF exchange in a GW calculation and the electron–hole interaction in a BSE calculation uses the random phase approximation (RPA) polarizability (Figure 3). Obtaining a good approximation to the ionization potential-electron affinity (IP-EA) gap is important in predicting particle–hole excitations accurately.

A BSE equation can be represented as
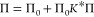
14where Π and Π_0_ are the interacting and noninteracting particle-hole propagators and K* is the proper interaction kernel. A BSE equation is solved as an eigenvalue problem in the form,
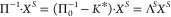
15by diagonalizing Π^–1^. *X*^*S*^ and Λ^*S*^ are eigenvectors and eigenvalues with an index, *S*. BSE calculations which use the GW@HF approximation with screening via the RPA polarizability (Π_0_ = Π_*RPA*_) overestimate excitation energies in C_60_ (Section III.B). If instead the bare electron–hole interaction is used in calculating the polarizability (Π_0_ = Π_*TDHF*_), poles of the polarizability shift to lower energy, and BSE excitation energies are underestimated. If this process is iterated once, then stronger screening in Π_*TDHF*_ reduces the pole shift to lower energy. This polarizability is denoted as Π_*scTDHF*_ as it is a partially self-consistent TDHF polarizability. This yields better agreement than either Π_*RPA*_ or Π_*TDHF*_ when used in calculating the IP-EA gap of C_60_ and the linear optical absorption spectrum in a BSE calculation (Sec. III B and III C). Continued iteration of this process would lead to self-consistency in the screened interaction (i.e., eigenvectors *X*^*S*^ and eigenvalues Λ^*S*^ used to construct the screened interaction), while retaining HF single-particle eigenvalues and wave functions in Table I in matrix elements in Π^–1^.

**Table I:** Inverse Polarizability Matrix Elements in Various BSE-TDA Approximations

method	matrix elements
DFT-RPA	ε_*a*_^*DFT*^–ε_*i*_^*DFT*^ + 2 (*ia*|*jb*)
HF-RPA	ε_*a*_^*HF*^–ε_*i*_^*HF*^ + 2 (*ia*|*jb*)
HF-scTDHF	ε_*a*_^*HF*^–ε_*i*_^*HF*^ + 2 (*ia*|*jb*) - (*ij*|*ab*)

Matrix elements used in these different approaches to polarizability and screening in GW/*BSE* calculations are summarized in Table I. In an HF-RPA or DFT-RPA calculation, the inverse polarizability contains HF or DFT single-particle energy differences, ε_*a*_ – ε_*i*_, where indices *i* and *a* denote occupied and virtual orbitals. The interaction kernel, K*, contains (*ia*|*jb*) terms corresponding to electron–hole pair hopping. In an HF-TDHF or HF-scTDHF calculation, K* also contains the electron–hole attraction terms, (*ij*|*ab*).

#### II.C.4 Oligothiophene-C_60_ Complex Number Densities

Number densities used for the T6-C_60_, T6-C_60_-T6 and T10-C_60_-T10 complex susceptibilities described below were 5.3 × 10^26^, 3.7 × 10^26^, and 2.7 × 10^26^ m^–3^, respectively, corresponding to a density of approximately 1070 kg m^–3^, the density of polythiophene. The number density of C_60_ was 1.4 × 10^27^ m^–3^, the density in bulk C_60_.^60^

## III Results

### III.A EA Spectra of BH_3_:NH_3_ Adduct and BH_3_ and NH_3_

**Figure 4:**
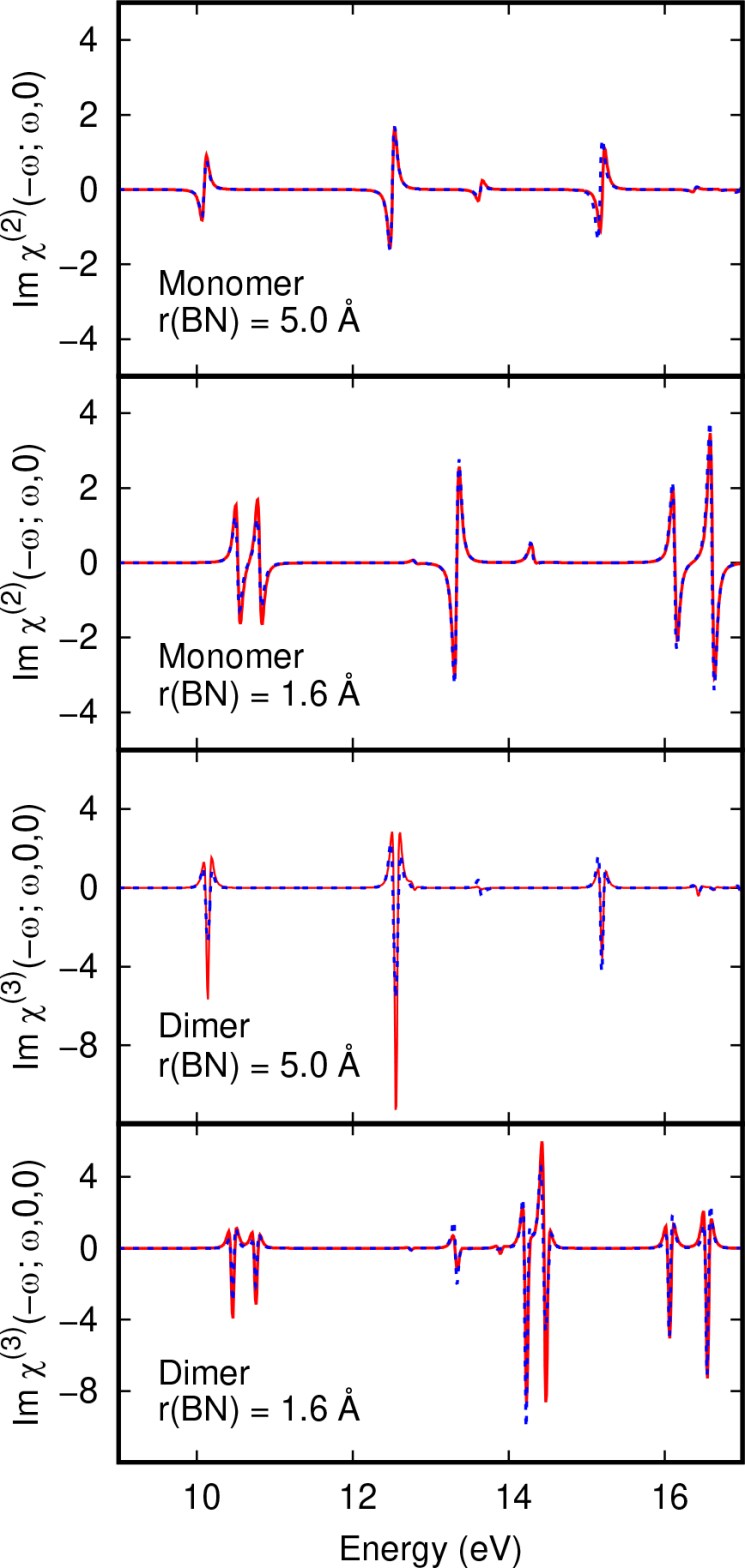
Comparison of Δ*A*(ω) spectra for the NH_3_BH_3_ adduct and separated NH_3_ and BH_3_ molecules and their dimers from finite field CIS calculations and nonlinear susceptibilities. (Top panels) NH_3_BH_3_ monomer with B–N distances of 5.0 and 1.6 Å. (Bottom panels) Dimers of NH_3_BH_3_ with B–N distances of 5.0 and 1.6 Å. Solid red lines show χ^(2)^ linear electrooptic spectra in units of 10^–21^ m^2^ V^–1^ and χ^(3)^ electroabsorption spectra in units of 10^–18^ m^2^ V^–1^. Dotted blue lines show finite field Δ*A*(ω) spectra with a field strength of 1.2 × 10^8^ V m^–1^.

Results of Δ*A*(ω) calculations for the BH_3_:NH_3_ adduct; BH_3_ and NH_3_ molecules with a BN separation of 5 Å; a pair of BH_3_:NH_3_ adducts and a pair of BH_3_ and NH_3_ molecules from FF calculations and perturbation theory expressions in eq 2 to 4 are presented in Figure 4. Molecular arrangements are shown in Figure 1. The top two panels of Figure 4 show absorption difference Δ*A*(ω) spectra for BH_3_ and NH_3_ molecules separated by 5 Å and a single BH_3_:NH_3_ adduct obtained from FF calculations and from the χ^(2)^ perturbative expression in eq 2. These are in very good agreement for both the equilibrium BH_3_:NH_3_ geometry, and when the molecules are separated by 5 Å.

For the latter, there are three strong first derivative lineshapes at 10.1, 12.5, and 15.2 eV. These are NσBσ* CT excitations mostly from the *H* – 0 level to the *L* + 0, *L* + 2 and *L* + 10 levels. Differences in excited and ground state dipole moments are 21.5, 24.9, and 13.1 D, respectively. Each transition has a nonzero transition dipole moment. Switching to the adduct equilibrium geometry, there are five strong first derivative lineshapes at 10.5, 10.8, 13.3, 16.1, and 16.5 eV. Differences in excited and ground state dipole moments are 2.8, 2.3, 10.1, 3.5, and 0.8 D, respectively. Magnitudues of dipole moment differences are smaller than for CT excitations, as expected.

**Figure 5:**
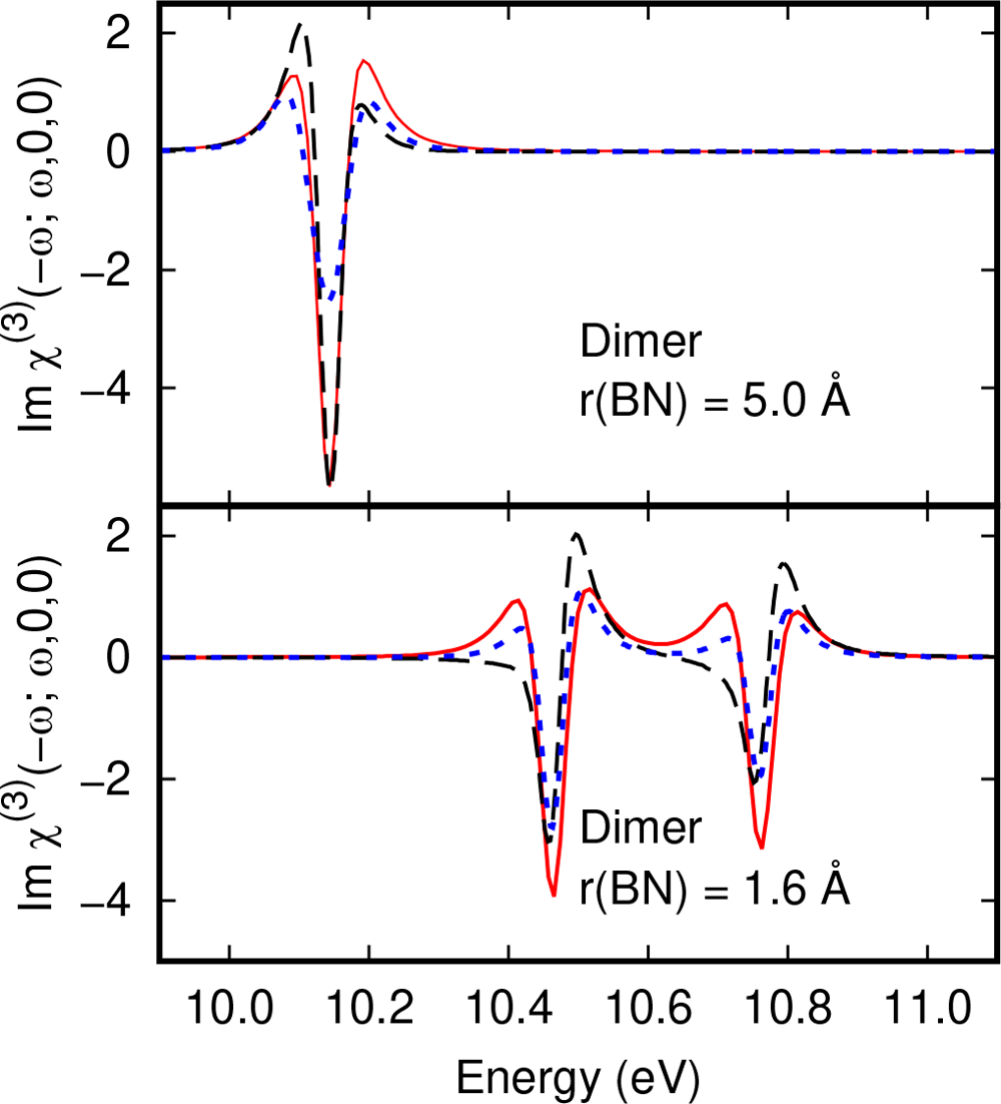
Dependence of finite field Δ*A*(ω) spectral lineshapes for NH_3_ BH_3_ dimers with a B–N separation of 5.0 or 1.6 Å on field strength compared to χ^(3)^(−ω; ω, 0, 0) in units of 10^–18^ m^2^ V^–1^ (solid red lines). Finite field spectra were obtained using a field strength of 1.2 × 10^8^ V m^–1^ (dotted blue lines) or 2.9 × 10^7^ V m^–1^ (dashed black lines).

Changes in the optical absorption spectrum of the FR and CT modes when an electric field is applied are illustrated in Figure 2. A pair of BH_3_:NH_3_ adducts has two FR modes in which the transition moments in either adduct are in phase (lower frequency, FR1 mode) or in antiphase (higher frequency, FR2 mode). For the mode at 10.5 eV in the adduct, the CIS energies in the adduct pair are 10.4586 and 10.4692 eV. The transition dipole moment connecting these states is 6.375 D. When an external electric field with strength 2.934 × 10^7^ V m^–1^ is applied, the −**μ**_12_·**F** interaction energy is 3.89 meV. Diagonalizing the 2 × 2 matrix in eq 10 predicts that these energies shift to 10.4572 and 10.4704 eV. This compares to 10.4578 and 10.4705 eV from the CIS calculation performed in a FF. The eigenvectors are (0.9503, −0.3113) and (0.3113, 0.9503) and thus there is relatively little mixing of the unperturbed excited states. Since the off-diagonal element, 3.89 meV, is small compared to the difference in diagonal elements, the difference in energies of FR1 and FR2 equal to 10.6 meV, limited mixing of FR1 and FR2 eigenvectors occurs when the static electric field is applied. Increasing the static electric field strength by a factor of 4 yields comparable diagonal and off-diagonal terms (10.6 vs 15.6 meV). Changes in FF Δ*A*(ω) spectral lineshapes which result from this change in static electric field strength are shown in the lower panel of Figure 5. When the lower strength field is applied, the lineshapes at both 10.5 and 10.8 eV are negative first derivative lineshapes. These change to second derivative lineshapes in the stronger static electric field and show much better agreement with the 2-photon term in the χ^(3)^ susceptibility in eq 10 plotted in the same graph. The peak at 10.8 eV in the adduct has an off-diagonal matrix element of 3.47 meV, and therefore, we expect similar behavior to that for the peak at 10.5 eV.

For the CT mode at 10.1 eV in the separated molecules, the local oscillator energies are 10.1425 and 10.1433 eV in the absence of an external electric field. In a field strength of 2.934 × 10^7^ V m^–1^ the −**μ**_12_·**F** interaction energy is 11.59 meV. Diagonalizing the 2 × 2 matrix for these CT1 and CT2 states yields energies of 10.1313 and 10.1545 eV, which compare to 10.1299 and 10.1540 eV from the CIS calculation performed in a FF. The eigenvectors are (0.7192, −0.6948) and (0.6948, 0.7192). Since the off-diagonal element, 11.59 meV, greatly exceeds the difference in diagonal elements, 0.8 meV, there is a full rotation of CT1 and CT2 eigenvectors in this case to the form given in eq 11 and 12. The change in splitting of the CT1 and CT2 levels is 22.4 meV. The effect of applying a four times stronger electric field is to increase the splitting of the lines without changing the dipole activities of the modes. The splitting at zero field strength is 1.8 meV, increasing to 24.1 and 96.4 meV in field strengths of 2.9 × 10^7^ V m^–1^ and 1.2 × 10^8^ V m^–1^. The limited change in FF EA spectral line shape is shown in the top panel of Figure 5. There is no increased dipole activity on increasing the field strength by a factor of 4; however, the line splitting has increased by a factor of 4.

From these results on the ammonia–borane model system, we conclude that there is excellent agreement between FF and perturbative calculations of second and third order nonlinear responses of asymmetric monomers and symmetric molecular dimers and that the Hamiltonian in eq 10 describes interactions of local oscillators very well.

### III.B C_60_ Quasiparticle Energy Levels and Excited States

**Figure 6:**
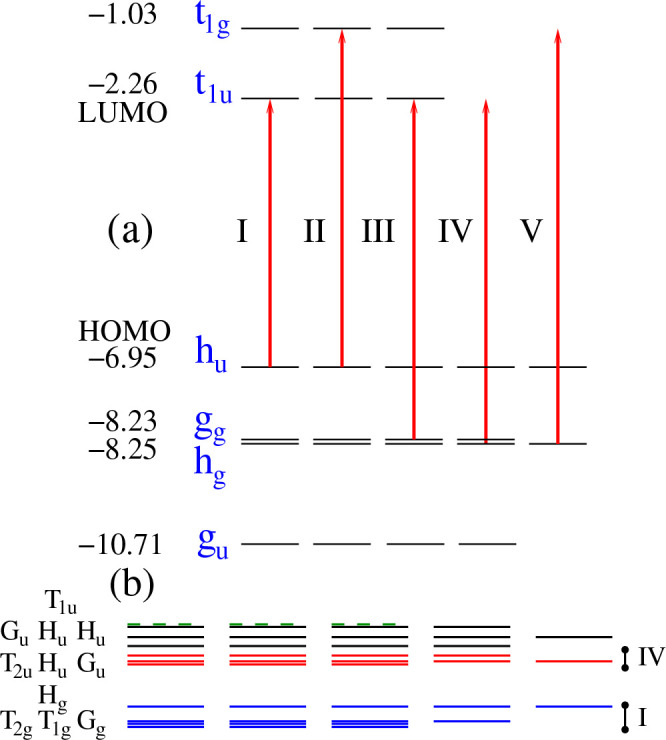
Schematic HF-scTDHF-GW energy level diagram and BSE-TDA singlet excited states of C_60_. (a) HF-scTDHF-GW frontier orbital energies in eV and MO symmetry labels. Principal optical transitions are indicated in roman numerals. (b) The first 11 singlet excited state energies of C_60_ from BSE-TDA with symmetries. Blue lines indicate transitions primarily from transition I and red lines indicate states primarily from transition IV. The first *T*_1*u*_ state is indicated by dotted green lines.

Predictions of the IP and EA of gas phase C_60_ from several GW^61−65^ and TDDFT^66^ calculations are available. The experimental IP^67^ and EA^68^ values in the gas phase are 7.6 and 2.7 eV, respectively. Single particle HF-scTDHF-GW energy levels and the first 11 BSE-TDA excited state energies of gas phase C_60_ are shown in Figure 6. Predictions of the IP, EA and IP-EA gap, E_*g*_, from various GW and TDDFT methods are compared to experiments in Table II. MOs shown belong to *t*_1*g*_, *t*_1*u*_, *h*_*u*_, *h*_*g*_, and *g*_*g*_ irreducible representations of the *I*_*h*_, icosahedral point group. The *h*_*u*_ HOMO and *t*_1*u*_ LUMO levels from the GW calculation with HF-RPA screening are at −7.35 and −1.82 eV with a gap of 5.43 eV. Using HF-scTDHF screening instead in the GW calculation, these levels are at −6.95 and −2.26 eV with a gap of 4.69 eV. A TD-ωB97X-D calculation yields a value, 5.44 eV, similar to that of GW@HF with RPA screening. These values compare to the experimental *E*_*g*_ of 4.9 eV. The former overestimates the gap by 10.8%, and the latter underestimates it by 4.3%.

**Table II:** Electron Affinity (EA), Ionization Potential (IP), and Energy Gap (*E*_*g*_) for C_60_ in the Gas Phase from HF, GW and TDDFT Methods and Experiment, in eV

	HF^a^	HF-RPA-GW^a^	HF-scTDHF-GW^a^	TD-ωB97X-D^b^	evGW^c^	GW^d^	scGW^e^	expt
EA	0.89	1.82	2.26	2.50	2.50	2.88	2.98	2.689 ± 0.008^f^
IP	7.87	7.35	6.95	7.94	7.41	7.23	7.86	7.6 ± 0.2^g^
E_*g*_	6.98	5.43	4.69	5.44	4.91	4.35	4.88	4.9

a This work/aug-cc-pVDZ.

b This work/def2-TZVP.

c Reference (62).

d Reference (63).

e Reference (61).

f Reference (68).

g Reference (67).

**Figure 7:**
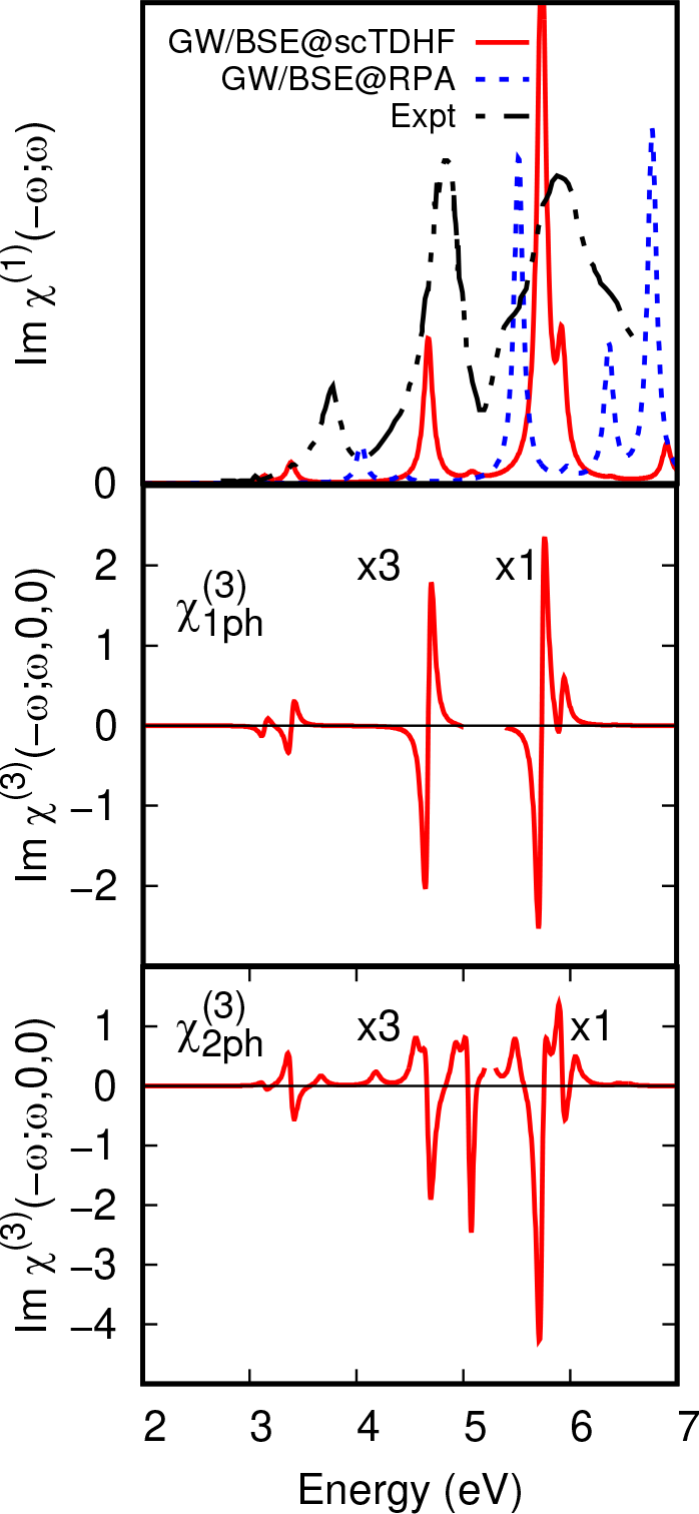
C_60_ χ^(1)^ optical absorption and EA χ_*xxxx*_^(3)^(−ω;ω,0,0) susceptibility. (Top panel) Optical absorption in C_60_ from GW/BSE calculations which used screened interactions calculated using screened TDHF (scTDHF) and RPA polarizabilites compared to experimental data from ref (35). (Middle panel) One photon term in χ_*xxxx*_^(3)^(−ω;ω,0,0). (Bottom panel) Two photon term in χ_*xxxx*_^(3)^(−ω;ω,0,0) in units of 1 × 10^–17^ m^2^ V^–1^. χ^(3)^ data were scaled ×3 below 5 eV.

Optical transition energies from CNDO/S^39^ and GW/BSE and TD-ωB97X-D calculations (this work), scGW/BSE^61^ and TD-BP98^37,69^ results are compared to experimental optical transition data in Table III. Optical absorption spectra from BSE calculations which used HF-RPA-GW or HF-scTDHF-GW electron and hole energies as input are compared to those from experiment in the top panel of Figure 7. Experimental optical absorption data are redrawn from Figure 1 in ref (35) and show the first (weak) ^1^*T*_1*u*_ level at 3.04 eV and three stronger absorptions at 3.78, 4.84, and 5.88 eV (labeled peaks C, D and E in ref (35)) and two weaker shoulders at 5.46 and 6.36 eV (labeled F and H). Electron–phonon coupling renders optically inactive modes active via a Herzberg–Teller mechanism and Leach and co-workers have attempted to assign these additional weak features in the spectrum.^35^ The initial assignment of lowest energy optical absorption features at 2.00 and 2.07 eV was to the lowest ^1^*T*_1*g*_ and ^1^*T*_2*g*_ transitions.^35^ However, further experiment and theoretical reanalysis concluded that the vibronic origins of the S1 and S2 states lie at 2.01 and 2.02 eV and are transitions to the 1^1^*T*_1*g*_ and 1^1^*G*_*g*_ levels.^40^

**Table III:** Excitation Energies for the First Ten Levels of All Symmetries and Optically Active ^1^*T*_1*u*_ Levels to around 6 eV in C_60_^k^

	CNDO/S^a^	RPA-BSE^b^^,^^c^	scTDHF-BSE^b^^,^^c^	scTDHF-BSE^b^^,^^d^	TD-ωB97X-D^b^^,^^e^	scGW^f^	TD-BP86^g^	TD-BP86^h^	expt^i^^,^^j^
1^1^*T*_2*g*_	2.29	2.06	1.53	1.63	2.09	1.92			
1^1^*T*_1*g*_	2.33	2.16	1.54	1.65	2.16	1.88			2.01
1^1^*G*_*g*_	2.34	2.22	1.55	1.66	2.18	1.82			2.02
1^1^*H*_*g*_	2.65	2.59	1.85	1.98	2.49	2.15			
1^1^*T*_2*u*_	2.76	3.12	2.51	2.50	3.14				
1^1^*H*_*u*_	3.01	3.24	2.56	2.56	3.28				
1^1^*G*_*u*_	3.11	3.26	2.65	2.66	3.38				
2^1^*G*_*u*_	3.38	3.65	2.80	2.84	3.87				
2^1^*H*_*u*_	3.70	3.89	2.94	3.00	4.01				
1^1^*T*_1*u*_	3.36	**4.04**	3.14	3.16	**4.06**		2.87	2.82	3.04
2^1^*T*_1*u*_	4.00	4.43	3.39	3.41	4.59		3.56	3.51	**3.78**
3^1^*T*_1*u*_	**4.26**	**5.51**	**4.66**	**4.71**	**5.74**		4.54	4.48	4.06
4^1^*T*_1*u*_	4.59	5.96	5.07	5.10	6.12		5.54	5.47	4.35
5^1^*T*_1*u*_	**4.97**	**6.36**	5.12	5.33			6.07	5.98	**4.84**
6^1^*T*_1*u*_	5.49	6.58	**5.73**	**5.79**					5.46
7^1^*T*_1*u*_	**5.57**	**6.76**	**5.91**	6.60					**5.88**
8^1^*T*_1*u*_	6.27	7.41	6.38	6.91					6.36

a Reference (39).

b This work.

c aug-cc-pVDZ.

d mod. def2-TZVP

e def2-TZVP.

f Reference (61).

g Reference (69).

h Reference (37).

i Reference (35).

j Reference (40).

k Transitions and experimental absorptions with large oscillator strength are shown in bold.

There is generally good agreement in predictions of optically inactive excited state energies from CNDO/S, GW/BSE and TD-ωB97X-D calculations and experiment. CNDO/S and BSE-TDA (this work) predict the ^1^*T*_2*g*_ state to be the lowest singlet excited state and to lie just below the ^1^*T*_1*g*_ state, in contrast to the reanalysis of experimental data mentioned above.^40^ These methods predict the same ordering for the first 9 excited states and good overall agreement in energies (Table III). scGW predicts the ^1^*G*_*g*_ level to be the lowest singlet excited state at 1.82 eV, followed by the ^1^*T*_1*g*_ and ^1^*T*_2*g*_ states at 1.88 and 1.92 eV. It was not possible to completely determine the symmetries of the first nine excitations predicted by TD-ωB97X-D. However, based on the level degeneracy and very good agreement with results of RPA-BSE calculations, it is likely that TD-ωB97X-D predicts the same ordering as the BSE methods used here. The effect of using a modified def2-TZVP basis versus an aug-cc-pVDZ basis is seen in columns four and five of Table III. Excitation energies are increased by up to 0.1 eV with the modified def2-TZVP basis, but otherwise, there is good agreement in excitation energies and state ordering.

Excited state calculations for C_60_ have mostly focused on the optically active n^1^T_1*u*_ states. These are compared in the bottom part of Table III. CNDO/S calculations predict eight ^1^*T*_1*u*_ states in the range of 3.36 to 6.27 eV. CNDO/S states for which there is a large oscillator strength from the ground state are in bold in Table III. Leach and co-workers assigned nine ^1^*T*_1*u*_ excited states on the basis of comparison to results of CNDO/S calculations.^39^ Experiment shows strong transitions at 3.78, 4.84, and 5.88 eV^35,36^ in *n*-hexane (Figure 7).

### III.C C_60_ Nonlinear Optical Absorption

First of all, we consider overall magnitudes of χ^(3)^ susceptibilities for C_60_. Susceptibilities in eq 2 to 4 contain the molecular number density as a prefactor. The value adopted for the prefactor for C_60_ (Section II.C.4) is the density found in bulk C_60_,^60^ 1.44 × 10^27^ m^–3^. The magnitude of the off-resonance 1*ph* line at 4.67 eV in C_60_ using this number density is 1.0 × 10^–22^ m^2^ V^–1^. Replacing two zero frequency denomimators in the χ^(3)^ expression in eq 3 on-resonance by a factor  for the line at 4.66 eV yields a resonant enhancement factor of 9 × 10^3^ and a magnitude of 9.0 × 10^–19^ m^2^ V^–1^, of the order of intensities of lines in Figure 7. The value of the line width parameter used was Γ = 0.04 eV. Distribution of oscillator strength over several vibrational excited states was not included and so this magnitude exceeds the usual expected resonant magnitude of third order nonlinear susceptibilities, which are typically of order 1.0 × 10^–21^ m^2^ V^–1^.^17^

The lowest dipole allowed excitations in C_60_ in the gas phase *I*_*h*_ point group symmetry lie about 1 eV above the S1 excited state (Table III). Intermediate states that contribute to the χ^(3)^ susceptibility in eq 3 and 4 must couple to states that are dipole coupled to the ^1^*A*_*g*_ ground state. In the *I*_*h*_ point group, Cartesian monomials *x*, *y* and *z* transform according to the *T*_1*u*_ ireducible representation (irrep). The only direct product with *T*_1*u*_ which includes *A*_*g*_ in the resulting direct sum is *T*_1*u*_ itself,
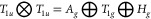
16Consequently, only *T*_1*u*_ states are dipole coupled to the ground state, intermediate states in the 2*ph* terms in eq 4 must belong to one of the *A*_*g*_, *T*_1*g*_, or *H*_*g*_ irreps in the absence of higher order couplings and the intermediate states in the 1*ph* terms in eq 3 must belong to *T*_1*u*_. As expected, χ_1*ph*_^(3)^ shows negative, first derivative lines, which are expected to vary in intensity as the square of intensity of the linear optical absorption intensity, owing to dependence on the same dipole matrix elements. Analysis of χ_2*ph*_^(3)^ also depends on dipole coupling between *T*_1*u*_ excited states and *A*_*g*_, *T*_1*g*_, and *H*_*g*_ excited states, as noted above. Strong second order resonances can occur when there is a state belonging to one of these irreps close in energy to a *T*_1*u*_ state.

### III.D Thiophene-C_60_ Linear and Nonlinear Optical Absorption

**Figure 8:**
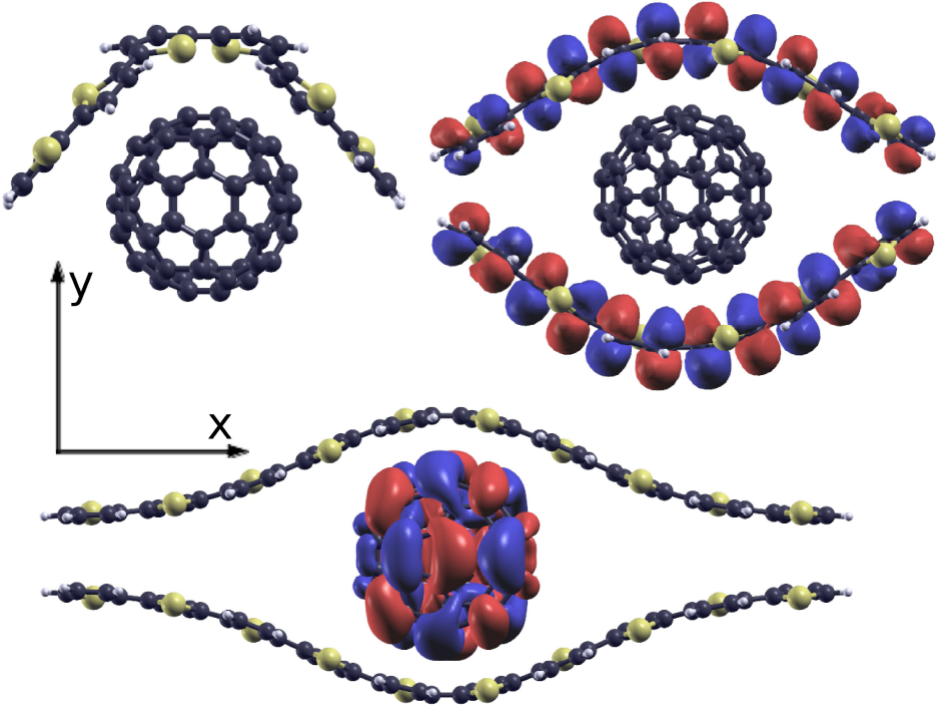
Structures of PT-C_60_ aggregates used in this work: (top left) T6-C_60_, (top right) T6-C_60_-T6, (Bottom) T10-C_60_-T10. Isosurfaces of the HOMO of T6-C_60_-T6 and the LUMO of T10-C_60_-T10 aggregates are also shown.

χ^(1)^, χ^(2)^, and χ^(3)^ linear and nonlinear optical absorption calculations were performed for a sexithiophene-C_60_ complex, T6-C_60_. Linear absorption and χ^(3)^ calculations were performed for similar complexes with two sexithiophenes (T6-C_60_-T6) and two decathiophenes (T10-C_60_-T10), each using the HF-scTDHF-BSE methods described in Section II.C.3. Structures of the complexes and two frontier orbital isosurfaces are shown in Figure 8. These structures represent the simplest conceivable contacts between donor and acceptor in this BHJ blend. In the absence of more detailed structural information from experiment they are the most appropriate structures for the current study.

Coordinates of the complexes are available in Tables S7 to S9. TD-ωB97X-D calculations of CT excited state energies were also performed for the T6-C_60_-T6 and T10-C_60_-T10 complexes. CT and thiophene oligomer and infinite polymer chain excited state energies are compared in Table IV.

**Table IV:** Energies of Principal CT and Polymer Chain (PC) Excited States in T6 and T10 Fragments Bound to C_60_ from GW/BSE and TDDFT Calculations, a Single Infinite Polythiophene Chain from a CIS Calculation, and P3[octyl]thiophene Condensed Polymer Experiment

state	T6-C_60_^a^	T6-C_60_-T6^a^	T6-C_60_-T6^b^	T10-C_60_-T10^a^	T10-C_60_-T10^b^	T10-C_60_^c^	PT	expt.
CT	1.92	1.98	1.38	2.14	1.15	1.69		0.99^d^
CT		2.05	1.43	2.23	1.49			
PC	2.88	2.95		2.75		2.62	2.78^e^	2.0^f^

a This work scTDHF-BSE.

b This work TD-ωB97X-D.

c GW/BSE. Reference (32).

d Reference (26).

e Reference (19).

f Reference (70).

For each complex, the first 10 excited states from HF-scTDHF-BSE are intramolecular C_60_ local excitons. They lie in ranges 1.74 to 1.85 (T6-C_60_), 1.64 to 1.83 (T6-C_60_-T6) and 1.83 to 2.00 eV (T10-C_60_-T10). These values compare to the ^1^*T*_2*g*_, ^1^*T*_1*u*_ and ^1^*G*_*g*_ excited state energies (1.63 to 1.66 eV) in C_60_ (Table III). In T6-C_60_, the next three states are the lowest CT states, which occur at 1.92, 1.94, and 1.98 eV, and consist primarily of transitions from the *H* + 0 level to the *L* + 0, *L* + 2 and *L* + 1 levels, respectively. There is a large change in permanent dipole moment in these excitations (19.2, 17.9, and 21.6 D, respectively). Transition moments connecting the ground state to the CT states at 1.92 and 1.94 eV are parallel to the *y* (charge transfer) direction and the transition moment to the state at 1.97 eV is parallel to the *x* direction (thiophene chain) direction. Low energy CT excited states and χ^(3)^ spectra for the two complexes with two oligothiophene strands bound to C_60_ are similar to those in T6-C_60_ with the addition of transitions from both the *H* – 0 and *H* – 1 levels. For T6-C_60_-T6 there are four states at 1.98, 2.00, 2.01, and 2.03 eV which are predominantly *H* – 0 and *H* – 1 to *L* + 0 and *L* + 2. Transition moments to these states are parallel to *y*. There are three further CT states at 2.05, 2.06, and 2.09 eV which are predominantly *H* – 0 and *H* – 1 to *L* + 1 and transition moments to these states are parallel to *x*. For T10-C_60_-T10, the pattern is the same as that for T6-C_60_-T6. Excitation energies and dipole matrix elements between states are given in Table S4. Multiple states in a small energy range can lead to nonadiabatic effects,^39,41^ but this is beyond the scope of the present work.

The first nine excited states of T6-C_60_-T6 and the first 12 excited states of T10-C_60_-T10 in the TD-ωB97X-D calculations are CT states. In T10-C_60_-T10 they are grouped in sixes as transitions from H-0 and H-1 to L+0, L+1 and L+2 (1.15 to 1.23 eV) and from H-2 and H-3 to L+0, L+1 and L+2 (1.49 to 1.63 eV).

Values of CT excited state energies from *scTDHF* – GW/*BSE* calculations compare to a CT excited state energy of 1.69 eV from a GW/*BSE* calculation on a T10-C_60_ complex^32^ and 0.99 eV from an estimate based on comparisons of experimental data^26^ or 1.05 eV in electroluminescence in a P3HT:PCBM blend.^25^ Omission of screening from the environment surrounding the complex and limited basis sets contribute to the difference in predicted CT energies and experiment. However, conclusions regarding the observability of CT states in these systems using EA spectroscopy are expected to remain valid as long as relative intensities of features from the oligothiophene and CT states and position of the CT state below the main thiophene absorption are correct.

**Figure 9:**
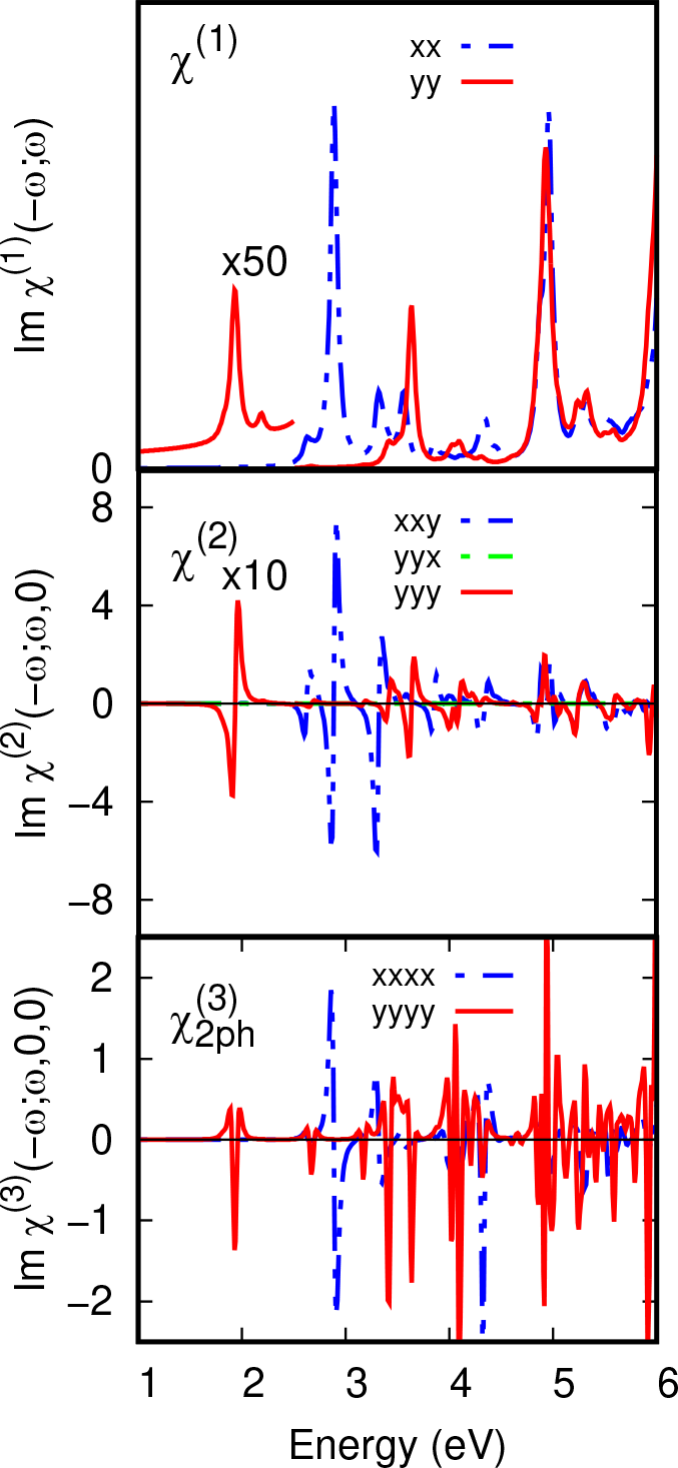
T6-C_60_ χ^(1)^(−ω; ω) optical absorption and χ^(2)^(−ω; ω, 0) and χ^(3)^(−ω; ω, 0, 0) nonlinear susceptibility elements. (Top panel) Optical absorption in T6-C_60_ for the optical field in the *x* (dashed blue line) and *y* (solid red line) directions indicated in Figure 7. The weak CT *yy* absorption at 1.9 eV is scaled ×50 below 2.5 eV. (Middle panel) χ^(2)^(−ω; ω, 0) elements with optical and static fields aligned with the *y* direction (*yyy*) and with the optical field in the *x* direction and static field in the *y* direction (*xxy*). χ^(2)^ elements are in units of 10^–20^mV^–1^. The *yyy* element is scaled ×10 below 2.5 eV. *xxx* and *yyx* elements are small and are not shown. (Bottom panel) χ^(3)^(−ω; ω, 0, 0) elements with optical and static fields aligned with the thiophene chain direction *x* and in the charge transfer direction *y*. χ^(3)^ elements are in units of 10^–17^ mV^–1^.

Linear absorption and *xxy*, *yxx*, and *yyy* elements of χ^(2)^ and *xxxx* and *yyyy* elements of χ^(3)^ susceptibilities for a single T6 oligomer bound to C_60_ with *C*_*2*_ symmetry are shown in Figure 9. The CT excited state contributes a first derivative line shape in the χ_*yyy*_^(2)^ spectrum, which has an intensity about 10% of the intensity of the signal from T6 in χ_*xxy*_^(2)^ at 2.88 eV, compared to about 1% in the linear absorption spectrum (Figure 9). The χ_*yyyy*_^(3)^ spectrum has a second derivative line shape at the CT energy (1.92 eV), which has an intensity about 50% of the T6 peak at 2.88 eV. In this case, the static and optical frequency fields are parallel. Linear and χ^(3)^ spectra for T6-C_60_-T6 and T10-C_60_-T10 and additional elements of the χ^(3)^ spectrum of T6-C_60_ are shown in Figures S2 to S4. Similar features are observed for each complex: The linear absorption spectrum is dominated by the main thiophene absorption around 2.8 eV (Table IV) polarized parallel to the *x* (thiophene chain) direction and an isotropic absorption at 4.8 eV from C_60_. One-photon χ^(3)^ spectra are dominated by the *xxxx* element at the main thiophene absorption energy, as expected. The χ_*xxyy*_^(3)^ and χ_*yyxx*_^(3)^ one- and two-photon spectra have little intensity at the CT energy (Figures S2 to S4).

**Figure 10:**
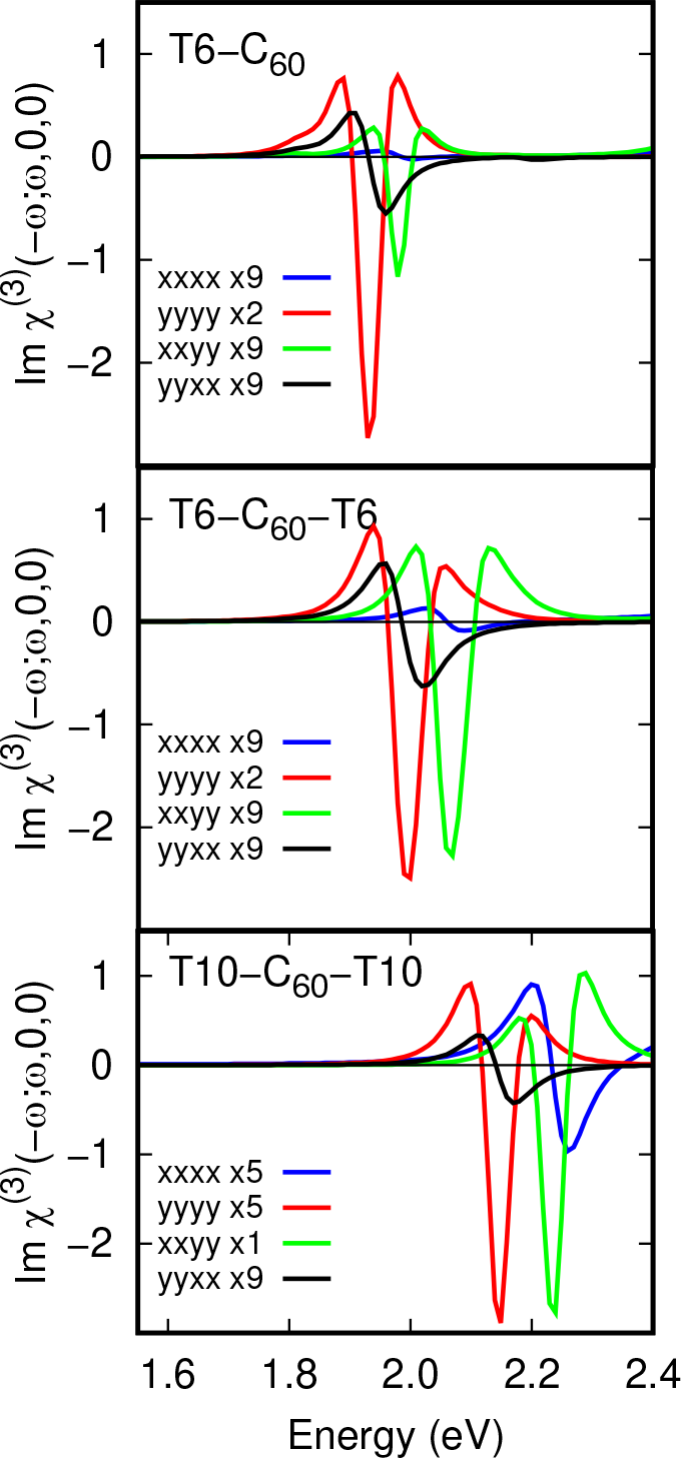
T6-C_60_, T6-C_60_-T6 and T10-C_60_-T10 complex χ^(3)^(−ω; ω, 0, 0) susceptibilities in an energy window around the CT excited state energy. (Top panel) *xxxx*, *yyyy*, *xxyy* and *yyxx* matrix elements for T6-C_60_ show first and second derivative lineshapes with varying intensities (note scaling factors in the key). The *yyyy* element is dominant and has both electric fields perpendicular to the T6 chain direction. (Middle panel) For the T6-C_60_-T6 complex the pattern of intensities is similar to that of T6-C_60_ with the *yyyy* element dominant. (Bottom panel) For the T10-C_60_-T10 complex the *xxyy* element is dominant with the static electric field perpendicular to the T10 chain direction and the optical frequency field parallel to the chain dirtection. χ^(3)^ elements are given in units of 10^–17^ mV^–1^.

Two-photon χ^(3)^ spectra for the three complexes studied in the CT excited state energy range are shown in Figure 10. These spectra contain either weak first derivative or stronger second derivative lineshapes, depending on the specific tensor element. Inspection of the top panel of Figure 10 shows first and second derivative features in the *yyxx* and *yyyy* elements of χ^(3)^ at 1.93 eV and first and second derivative features in the *xxxx* and *xxyy* elements of χ^(3)^ at 1.98 eV for T6-C_60_. The second derivative *yyyy* line shape is dominant.

Dipole couplings between the ground and CT excited states and between CT excited states are given in Tables S4 to S6. For T6-C_60_ this shows large permanent moments for each CT excited state and weak couplings between the excited states. The pattern of zero and nonzero dipole matrix elements shows that the first two CT states belong to the *A* (even) irrep and the third belongs to the *B* (odd) irrep with respect to C_2_ rotations about the *y* axis. Both of these contribute to the strong *yyyy* second derivative line shape at 1.93 eV, which is approximately 10 times stronger than the *xxyy* line at 1.97 eV.

When second oligothiophenes are introduced in the T6-C_60_-T6 and T10-C_60_-T10 complexes, the number of low energy CT states increases from three to seven. The pattern of dipole couplings between the ground and excited states is similar to the T6-C_60_ complex, but there are additional strong off-diagonal dipole couplings between excited states 1 and 3 and 2 and 4 in T6-C_60_-T6 (Table S5) and between states 1 and 2 and 3 and 4 in T10-C_60_-T10 (Table S6). This leads to increases in the intensity of the *xxyy χ*^(3)^ element relative to *yyyy* in T6-C_60_-T6 and in T10-C_60_-T10 *xxyy* is the dominant element, by a factor of 5. Thus, χ^(3)^(−ω; ω, 0, 0) matrix elements are sensitive to the molecular structure in oligothiophene/C_60_ complexes. Furthermore, the strength of the signal from a CT state in a thiophene-C_60_ complex increases relative to the main thiophene linear optical absorption peak on going from χ^(1)^ to χ^(2)^ by a factor of 10 or so and by a factor of 50 on going to the χ^(3)^ response. EA spectroscopy therefore offers a means of significantly enhancing relative strengths of optical absorption signals which are weak in linear spectroscopies.

## IV Discussion and Conclusions

### IV.A Observing CT States in Experiment

Reviews by Piliego and Loi,^1^ Gao and Inganäs,^2^ and Vandewal^3^ cite direct observation of CT state absorption and emission by C_60_ polymer blends. The kinetics of CT excited state dissociation into free carriers in bilayer devices consisting of C_60_ and trimethine cyanine dye (Cy3-P) have been measured in an external field of 5 × 10^7^V m^–1^ by Devižis and co-workers^29^ using a dynamic EA spectroscopy technique. Here the dissociation was inferred by monitoring the EA spectrum in the range 400 to 650 nm (3.1 to 1.9 eV) and it was assumed that free carrier generation screened the external, static electric field, resulting in a reduction of the measured EA spectral intensity. The lowest energy EA feature in the pure Cy3-P spectrum just above 2 eV was also found in the C_60_ bilayer device but no new feature in the bilayer device which might be attributed to a CT state is clearly evident. As noted above, CT states in conjugated polymer systems containing C_60_ derivatives^26^ typically lie in the range 1.0 to 1.6 eV.

As shown in Figure 9, EA nonlinear optical spectroscopy for CT states in this BHJ system increases the relative sensitivity to the CT state compared to linear optical absorption by around 50 times. EA spectroscopy may therefore offer a means of identifying the energies of CT excited states and measuring their decay kinetics. There have been attempts to identify weak features observed in various spectroscopies in the sub-band gap region of conjugated polymer–fullerene BHJ devices. Beenken and co-workers found a peak in PDS measurements at 1.6 eV in P3HT:PCBM films but concluded that it was impossible to distinguish between a CT state or polaron transition in assigning this feature.^10^

### IV.B Summary

We have investigated prospects for spectroscopic identification of CT excited states in the sub-band gap region of thiophene–fullerene BHJ systems using CIS, GW/BSE and TD-ωB97X-D calculations on a model system consisting of pairs of BH_3_:NH_3_ molecules, gas phase C_60_ and three thiophene-C_60_ complexes.

CIS calculations on BH_3_:NH_3_ and BH_3_:NH_3_ dimers as adducts and separated molecules were used to demonstrate excellent agreement between finite field calculations of the change in absorption spectrum, Δ*A*(ω), and imaginary parts of the second and third order sum over states perturbation theory expressions of Orr and Ward.^16^ This is important for establishing confidence in the results of perturbative calculations when applied to larger systems where convergence of finite field calculations to the required precision is difficult. A single BH_3_:NH_3_ molecular pair lacks inversion symmetry, and the leading nonlinear response is at second order. Shifts in transition energies and absorption intensities are very well reproduced by a 2 × 2 model Hamiltonian applied to the centrosymmetric cases in which excited state transition energies are on the diagonal, and −**μ**_12_·**F** dipolar coupling between excited states and an external electric field are the off-diagonal elements. Typically one state is optically bright and one is dark. When the magnitude of the off-diagonal matrix element exceeds the difference in bright and dark state transition energies the new eigenstates mix the bright and dark states, resulting in two optically active lines and an overall second derivative line shape for the change in optical absorption.

Optical absorption spectra for C_60_ were calculated using GW/BSE with a SCF HF starting point and TD-ωB97X-D. When screening in the BSE calculations on C_60_ was introduced using a screened interaction based on an RPA polarizability, optical absorption energies are considerably overestimated, and relative intensities of several peaks in experiment are not reproduced. TD-ωB97X-D yielded excitation energies similar to those in the GW/BSE calculation with the RPA polarizability. However, when a partly self-consistent polarizability denoted scTDHF which includes ladder terms is used instead, there is much better agreement with experiment for both optical absorption energies and relative intensities. GW/BSE excited state energies and wave functions from this method are used to calculate χ^(3)^ for gas phase C_60_.

Finally, GW/BSE calculations were performed for three thiophene-C_60_ complexes using GW/BSE and scTDHF polarizability. In each case, these calculations predict several CT excited state energies which cluster together in a narrow energy range. The origin of this close spacing of excited states and dipole coupling is excitation from the thiophene HOMO level into the LUMO, LUMO+1 and LUMO+2 levels. It is concluded that EA spectroscopy on conjugated polymer–fullerene systems could be used to identify energies of CT excited states in BHJ devices.

## Supporting Information Available

Comparison of linear absorption χ^(1)^ and nonlinear absorption χ^(3)^ spectra for C_60_ from aug-cc-pVDZ and modified def2-TZVP basis sets; linear absorption χ^(1)^ and nonlinear absorption χ^(3)^ spectra for the T6-C_60_, T6-C_60_-T6, and T10-C_60_-T10 complexes shown in Figure 8; tables of modified basis sets used for thiophene/C_60_ complexes; permanent dipole moments of the ground state and CT excited states of these complexes and transition dipole moments connecting these states; and Cartesian coordinates for each of the thiophene-C_60_ complexes (PDF)
