# Supplementary material for: Spectroscopic Identification of the Charge Transfer State in Thiophene/Fullerene Heterojunctions: Electroabsorption Spectroscopy from GW/BSE Calculations
Source: J Phys Chem C Nanomater Interfaces. 2023 Aug 4;127(32):15928–42. doi: 10.1021/acs.jpcc.3c03734 (PMC10440814; doi:10.1021/acs.jpcc.3c03734)
Supplement: Supplementary file 1 — jp3c03734_si_001.pdf [file jp3c03734_si_001.pdf]

Supporting Information for Spectroscopic Identification of the Charge Transfer State in Thiophene/Fullerene Heterojunctions: Electroabsorption Spectroscopy from GW/BSE Calculations

Smruti Ranjan Sahoo and Charles H. Patterson*

School of Physics, Trinity College Dublin, Dublin 2, D02 PN40, Ireland

E-mail: Charles.Patterson@tcd.ie   sahoos@tcd.ie

This document contains: a comparison of linear absorption Im $\chi^{(1)}$ and nonlinear absorption Im $\chi^{(3)}$ spectra for C_{60} from aug-cc-pVDZ and modified def2-TZVP basis sets; linear absorption Im $\chi^{(1)}$ and nonlinear absorption Im $\chi^{(3)}$ spectra for the T6-C_{60}, T6-C_{60}-T6 and T10-C_{60}-T10 complexes shown in Fig. 8; tables of modified basis sets used for thiophene/C_{60} complexes; permanent dipole moments of the ground state and CT excited states of these complexes and transition dipole moments connecting these states; Cartesian coordinates for each of the thiophene-C_{60} complexes.

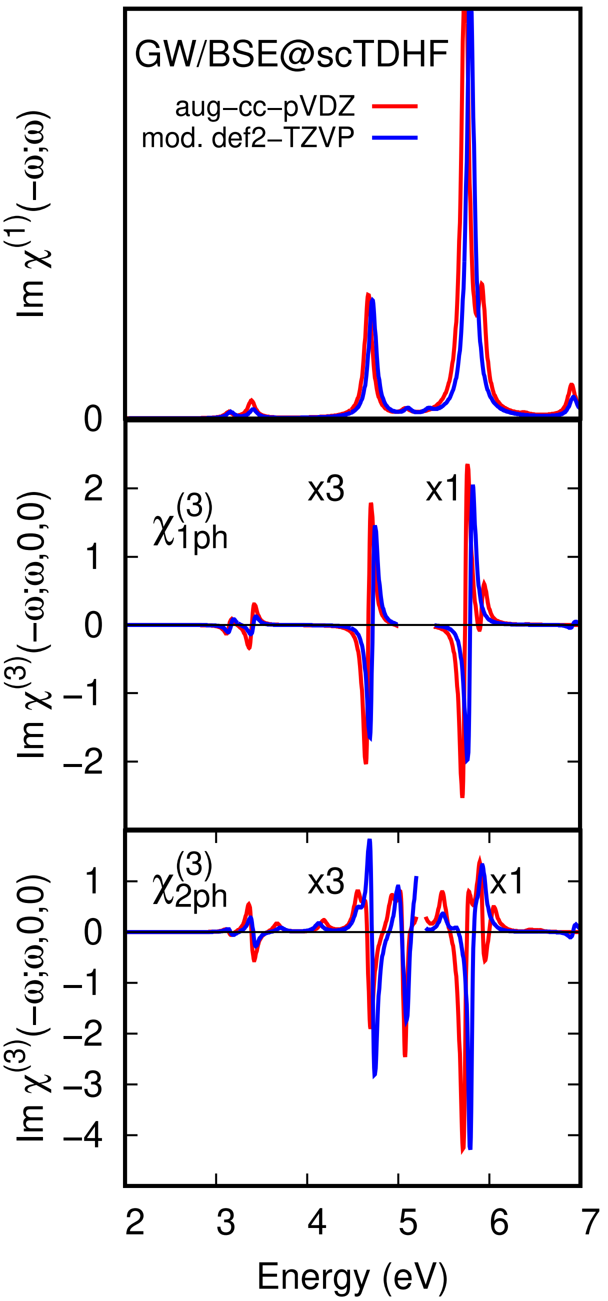

Figure S1: Comparison of Im $\chi^{(1)}(-\omega;\omega)$ optical absorption and Im $\chi^{(3)}(-\omega;\omega,0,0)$ susceptibility in C_{60} using an aug-cc-pVDZ basis (red line) and modified def2-TZVP basis (blue line). (Top panel) Optical absorption, (Middle panel) One photon term in Im $\chi^{(3)}_{xxxx}(-\omega;\omega,0,0)$. (Bottom panel) Two photon term in Im $\chi^{(3)}_{xxxx}(-\omega;\omega,0,0)$ in units of 1x10^{-17}m$^2V^{-1}$. Im $\chi^{(3)}$ data were scaled x3 below 5 eV.

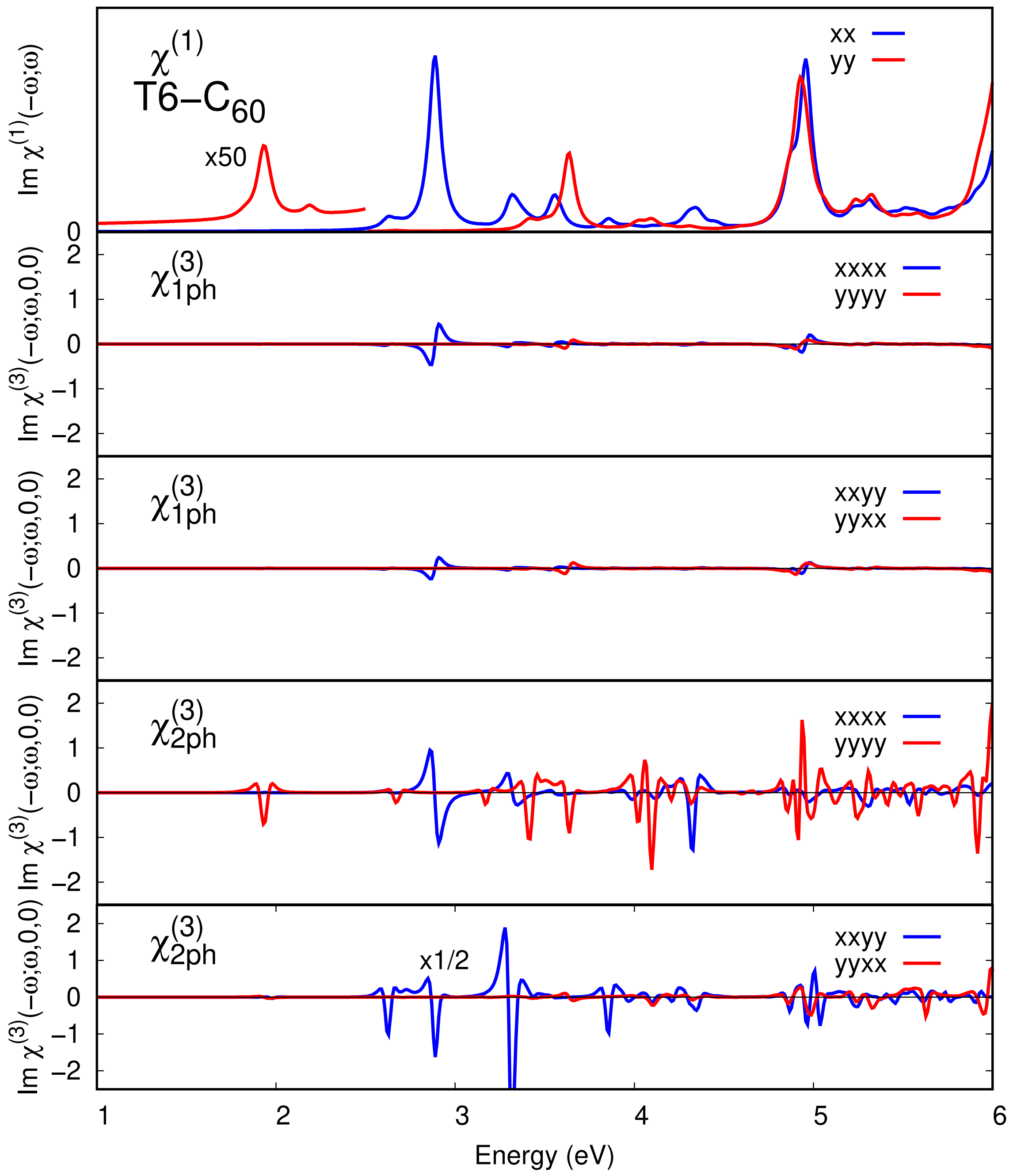

Figure S2: T6-C_{60} Im $\chi^{(1)}(-\omega;\omega)$ optical absorption and Im $\chi^{(3)}(-\omega;\omega,0,0)$ susceptibility elements. (Top panel) Optical absorption in T6-C_{60} for the optical field in the x (blue line) and y (red line) directions indicated in Fig. 8. The weak CT yy absorption at 1.9 eV is scaled x50 below 2.5 eV. (Second and third panels) One photon elements in Im $\chi^{(3)}(-\omega;\omega,0,0)$. (Fourth and fifth panels) Two photon elements in Im $\chi^{(3)}(-\omega;\omega,0,0)$. Im $\chi^{(3)}$ elements are in units of 10^{-17}mV^{-1}.

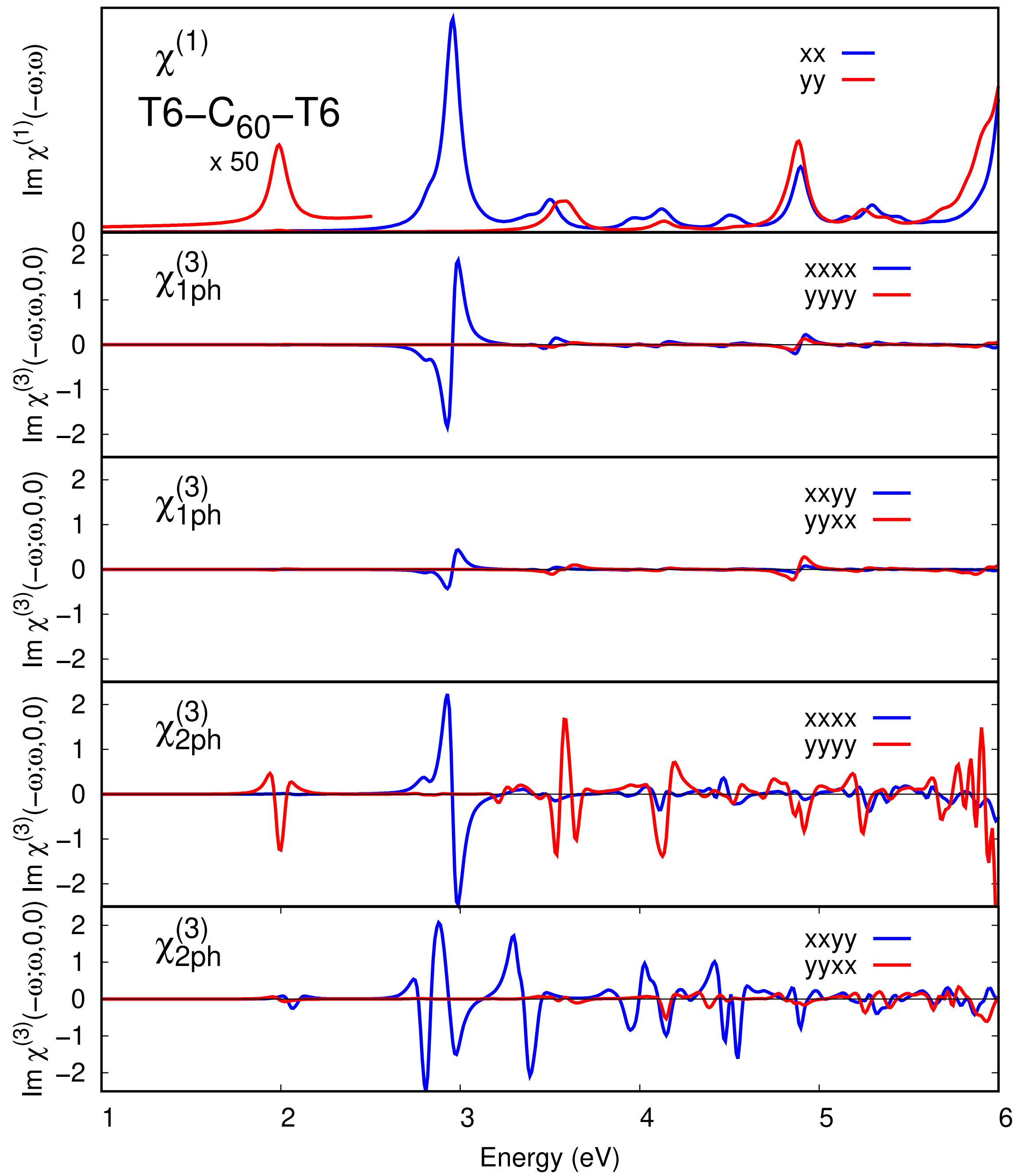

Figure S3: T6-C_{60}-T6 Im $\chi^{(1)}(-\omega;\omega)$ optical absorption and Im $\chi^{(3)}(-\omega;\omega,0,0)$ susceptibility elements. (Top panel) Optical absorption in T6-C_{60}-T6 for the optical field in the x (blue line) and y (red line) directions indicated in Fig. 8. The yy linear absorption spectrum is scaled x50 below 2.5 eV and contains the weak CT absorption at 1.98 eV. (Second and third panels) One photon elements in Im $\chi^{(3)}(-\omega;\omega,0,0)$. (Fourth and fifth panels) Two photon elements in Im $\chi^{(3)}(-\omega;\omega,0,0)$. Im $\chi^{(3)}$ elements are in units of $10^{-17}\mathrm{mV}^{-1}$.

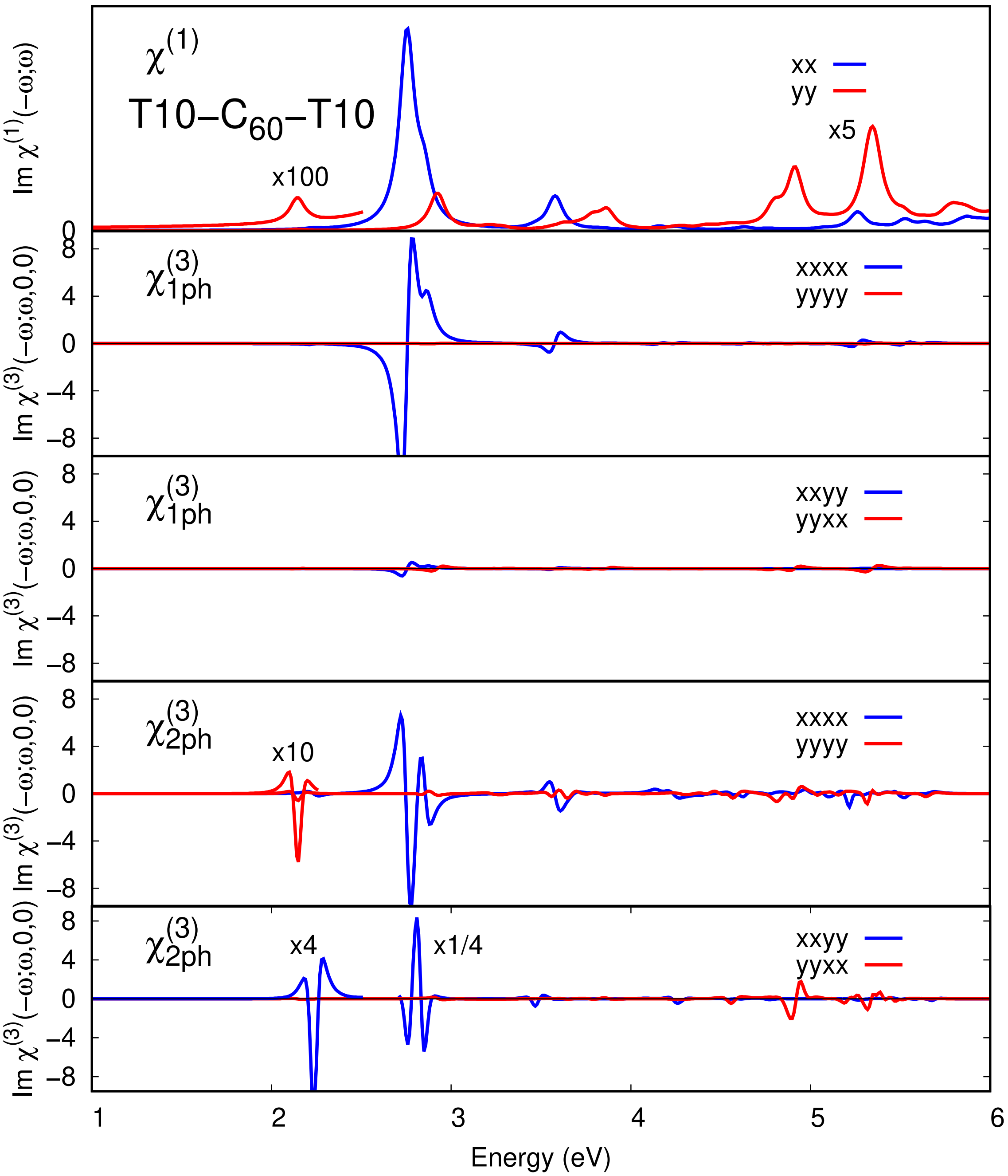

Figure S4: T10-C_{60}-T10 Im $\chi^{(1)}(-\omega;\omega)$ optical absorption and Im $\chi^{(3)}(-\omega;\omega,0,0)$ susceptibility elements. (Top panel) Optical absorption in T10-C_{60}-T10 for the optical field in the x (blue line) and y (red line) directions indicated in Fig. 8. The weak CT yy absorption at 2.14 eV is scaled x100 below 2.5 eV. (Second and third panels) One photon elements in Im $\chi^{(3)}(-\omega;\omega,0,0)$. (Fourth and fifth panels) Two photon elements in Im $\chi^{(3)}(-\omega;\omega,0,0)$. The Im $\chi^{(3)}_{yyyy}(-\omega;\omega,0,0)$ element shows a CT feature at 2.14 eV and is scaled x10 below 2.5 eV. Im $\chi^{(3)}$ elements are in units of $10^{-17}\mathrm{mV}^{-1}$.

Table S1: Modfied def2-SZVP basis set for hydrogen[1]. Exponent changes are highlighted in bold text (see text).

Shell	Exponent	Coefficient
S		
	13.0100000	0.00602519
	1.9620000	0.04502109
	0.4446000	0.20189726
S		
	**0.220000**	1.0000000
P		
	**0.720000**	1.0000000

Table S2: Modfied def2-TZVP basis set for carbon[1]. Exponent changes are highlighted in bold and omitted exponents are highlighted in bold red text (see text).

Shell	Exponent	Coefficient
S		
	13575.3496820	0.00022245814
	2035.2333680	0.00172327382
	463.2256235	0.00892557153
	131.2001959	0.03572798450
	42.8530158	0.11076259931
	15.5841857	0.24295627626
S		
	6.2067138	0.41440263448
	2.5764896	0.23744968655
S		
	0.5769633	1.0000000
S		
	**0.2000000**	1.0000000
S		
	**0.0951644**	1.0000000
P		
	34.6972322	0.00533336578
	7.9582622	0.03586410909
	2.3780826	0.14215873329
	0.8143320	0.34270471845
P		
	**0.5000000**	1.0000000
P		
	**0.2000000**	1.0000000
D		
	**0.8000000**	1.0000000
D		
	**0.3180000**	1.0000000
F		
	**0.7610000**	1.0000000

Table S3: Modfied 6-311G* basis set for sulfur.[2,3] Exponent changes are highlighted in bold text and omitted exponents are highlighted in bold red text.

Shell	Exponent	Coefficient
S		
	93413.4000	0.000743000
	13961.7000	0.005793000
	3169.91000	0.029954000
	902.456000	0.119028000
	297.158000	0.368432000
	108.702000	0.577299000
S		
	108.702000	0.143186000
	43.1553000	0.624465000
	18.1079000	0.283366000
S		
	5.56009000	1.000000000
S		
	2.13183000	1.000000000
S		
	0.42040300	1.000000000
S		
	**0.2236045**	1.000000000
P		
	495.040000	0.008309000
	117.221000	0.064024000
	37.7749000	0.277614000
	14.0584000	0.745076000
P		
	5.56574000	0.613712000
	2.26297000	0.443818000
P		
	0.80799400	1.000000000
P		
	0.27746000	1.000000000
P		
	**0.0771410**	1.000000000
D		
	0.650000000	1.000000000

Table S4: Excited State Energies in eV and Transition Dipole Moments in Debye Between Ground State (0) and CT Excited States (1-3) of the T6-C_{60} complex which has $C_2(y)$ Symmetry. Diagonal elements are permanent dipole moments. Upper and lower parts of the Table contain x and y components of dipole moments.

Energy (eV)	State	0	1	2	3
0.00	0	0.00	0.00	0.00	0.00
1.92	1		0.00	0.00	−0.18
1.94	2			0.00	−0.77
1.98	3				0.00
0.00	0	−0.59	−0.43	0.48	0.00
1.92	1		−19.20	1.28	0.00
1.94	2			−17.89	0.00
1.98	3				−21.62

Table S5: Excited State Energies in eV and Transition Dipole Moments in Debye Between Ground (0) and CT Excited States (1-7) of the T6-C_{60}-T6 complex which has $C_2(y)$ Symmetry. Diagonal elements are permanent dipole moments. Upper and lower parts of the Table contain x and y components of dipole moments.

Energy (eV)	State	0	1	2	3	4	5	6	7
0.00	0	0.00	0.00	0.00	0.00	0.00	0.34	0.45	0.21
1.98	1		0.00	0.00	0.00	0.00	−0.51	0.33	0.06
2.01	2			0.00	0.00	0.00	−0.78	−0.94	−0.34
2.01	3				0.00	0.00	−0.41	0.08	0.05
2.03	4					0.00	−0.31	0.07	−1.61
2.05	5						0.00	0.00	0.00
2.06	6							0.00	0.00
2.09	7								0.00
0.00	0	−0.21	0.98	0.00	0.44	0.14	0.00	0.00	0.00
1.98	1		16.10	1.03	−13.14	0.74	0.00	0.00	0.00
2.01	2			11.40	3.51	10.67	0.00	0.00	0.00
2.01	3				−15.60	1.63	0.00	0.00	0.00
2.03	4					−7.50	0.00	0.00	0.00
2.05	5						9.41	8.51	8.92
2.06	6							6.10	14.40
2.09	7								−15.32

Table S6: Excited State Energies in eV and Transition Dipole Moments in Debye Between Ground (0) and CT Excited States (1-7) of the T10-C_{60}-T10 complex which has $C_2(y)$ Symmetry. Diagonal elements are permanent dipole moments. Upper and lower parts of the Table contain x and y components of dipole moments.

Energy (eV)	State	0	1	2	3	4	5	6	7
0.00	0	0.00	0.00	0.00	0.00	0.00	0.00	0.00	0.00
2.14	1		0.00	0.00	0.00	0.00	0.01	−0.30	−0.74
2.15	2			0.00	0.00	0.00	−0.09	−0.74	0.34
2.17	3				0.00	0.00	0.49	0.17	0.06
2.18	4					0.00	−0.14	0.53	−0.05
2.23	5						0.00	0.00	0.00
2.23	6							0.00	0.00
2.25	7								0.00
0.00	0	−0.08	0.62	−0.15	−0.09	0.02	0.00	0.00	0.00
2.14	1		−11.07	−19.10	−0.23	−1.00	0.00	0.00	0.00
2.15	2			10.79	1.63	−0.22	0.00	0.00	0.00
2.17	3				−16.40	−13.58	0.00	0.00	0.00
2.18	4					16.05	0.00	0.00	0.00
2.23	5						−4.06	−16.03	0.52
2.23	6							−2.03	−17.14
2.25	7								5.93

Table S7: Cartesian Coordinates for T6-C_{60} Complex with $C_2(y)$ Symmetry in Å

6	3.5416780000	0.0137860000	1.6972510000
6	−3.5416780000	−0.0137860000	1.6972510000
6	3.2621980000	1.3751030000	1.6975180000
6	−3.2621980000	−1.3751030000	1.6975180000
6	0.6978460000	−3.4044800000	1.0029520000
6	−0.6978460000	3.4044800000	1.0029520000
6	0.6794410000	0.1386700000	−1.7649570000
6	−0.6794410000	−0.1386700000	−1.7649570000
6	1.1552510000	1.4346470000	−1.3224690000
6	−1.1552510000	−1.4346470000	−1.3224690000
6	0.2493420000	2.3974730000	−0.8952300000
6	−0.2493420000	−2.3974730000	−0.8952300000
6	0.5436950000	3.1990190000	0.2779290000
6	−0.5436950000	−3.1990190000	0.2779290000
6	0.6969570000	−3.4029520000	2.3917340000
6	−0.6969570000	3.4029520000	2.3917340000
6	0.6803110000	0.1389040000	5.1682170000
6	−0.6803110000	−0.1389040000	5.1682170000
6	1.6266470000	−0.8659990000	4.7200160000
6	−1.6266470000	0.8659990000	4.7200160000
6	1.6248050000	−0.8654430000	−1.3193660000
6	−1.6248050000	0.8654430000	−1.3193660000
6	1.1555060000	1.4341750000	4.7194430000
6	−1.1555060000	−1.4341750000	4.7194430000
6	1.1719550000	−2.1057780000	4.2900480000
6	−1.1719550000	2.1057780000	4.2900480000
6	1.1719920000	−2.1066890000	−0.8938860000
6	−1.1719920000	2.1066890000	−0.8938860000
6	0.2498530000	2.3959070000	4.2900350000
6	−0.2498530000	−2.3959070000	4.2900350000
6	1.7576000000	−2.7283040000	3.1171090000
6	−1.7576000000	2.7283040000	3.1171090000
6	1.7593990000	−2.7306590000	0.2763720000
6	−1.7593990000	2.7306590000	0.2763720000
6	0.5435860000	3.1971400000	3.1168150000
6	−0.5435860000	−3.1971400000	3.1168150000
6	2.7730860000	−2.0836050000	2.4221950000
6	−2.7730860000	2.0836050000	2.4221950000
6	2.7756470000	−2.0856860000	0.9704760000
6	−2.7756470000	2.0856860000	0.9704760000
6	1.7307830000	3.0015280000	2.4227890000
6	−1.7307830000	−3.0015280000	2.4227890000
6	1.7313090000	3.0028800000	0.9719940000

Continued on next page

6	−1.7313090000	−3.0028800000	0.9719940000
6	2.3955620000	1.2297450000	3.9944420000
6	−2.3955620000	−1.2297450000	3.9944420000
6	2.6880150000	−0.1923370000	−0.5990030000
6	−2.6880150000	0.1923370000	−0.5990030000
6	2.3969030000	1.2307690000	−0.5990850000
6	−2.3969030000	−1.2307690000	−0.5990850000
6	2.6866920000	−0.1918050000	3.9943960000
6	−2.6866920000	0.1918050000	3.9943960000
6	3.2470400000	−0.7869520000	2.8710450000
6	−3.2470400000	0.7869520000	2.8710450000
6	2.6781560000	1.9977040000	0.5239370000
6	−2.6781560000	−1.9977040000	0.5239370000
6	3.2502040000	−0.7884400000	0.5228160000
6	−3.2502040000	0.7884400000	0.5228160000
6	2.6766190000	1.9959990000	2.8708970000
6	−2.6766190000	−1.9959990000	2.8708970000
1	6.7058060000	−7.1181700000	1.4564500000
1	−6.7058060000	7.1181700000	1.4564500000
6	4.0844640000	−4.5886250000	−1.8458730000
6	−4.0844640000	4.5886250000	−1.8458730000
1	2.7156140000	−6.2452700000	−2.0723310000
1	−2.7156140000	6.2452700000	−2.0723310000
16	5.1882490000	−6.6909260000	−0.4103950000
16	−5.1882490000	6.6909260000	−0.4103950000
6	2.3037540000	−4.4971250000	−3.3469240000
6	−2.3037540000	4.4971250000	−3.3469240000
6	2.8331220000	−3.2436490000	−3.5549740000
6	−2.8331220000	3.2436490000	−3.5549740000
6	3.0087830000	−5.2543139999	−2.3878470000
6	−3.0087830000	5.2543139999	−2.3878470000
1	1.4136270000	−4.8465100000	−3.8504860000
1	−1.4136270000	4.8465100000	−3.8504860000
16	0.5431340000	−2.0507110000	−4.4930680000
16	−0.5431340000	2.0507110000	−4.4930680000
6	5.7569090000	−4.2299180000	0.0262100000
6	−5.7569090000	4.2299180000	0.0262100000
6	4.9852160000	−5.0108410000	−0.8035500000
6	−4.9852160000	5.0108410000	−0.8035500000
6	6.2817170000	−6.3245660000	0.8640040000
6	−6.2817170000	6.3245660000	0.8640040000
6	6.4953170000	−4.9803500000	0.9736240000
6	−6.4953170000	4.9803500000	0.9736240000
1	7.1490800000	−4.5404870000	1.7124600000
1	−7.1490800000	4.5404870000	1.7124600000

Continued on next page

1	5.7654850000	−3.1507550000	−0.0272100000
1	−5.7654850000	3.1507550000	−0.0272100000
16	4.2410640000	−3.0132830000	−2.5600410000
16	−4.2410640000	3.0132830000	−2.5600410000
6	1.9124970000	0.0537860000	−5.0127290000
6	−1.9124970000	−0.0537860000	−5.0127290000
6	0.6079210000	−0.3868760000	−4.9914730000
6	−0.6079210000	0.3868760000	−4.9914730000
6	2.2634660000	−2.1247930000	−4.2586110000
6	−2.2634660000	2.1247930000	−4.2586110000
6	2.8434290000	−0.9235940000	−4.6019240000
6	−2.8434290000	0.9235940000	−4.6019240000
1	2.1821960000	1.0697440000	−5.2639000000
1	−2.1821960000	−1.0697440000	−5.2639000000
1	3.9053880000	−0.7423080000	−4.5171050000
1	−3.9053880000	0.7423080000	−4.5171050000

Table S8: Cartesian Coordinates for T6-C_{60}-T6 Complex with $C_2(y)$ Symmetry in$\AA$

6	0.3582430000	−3.5252000000	0.0459570000
6	−0.3582430000	3.5252000000	0.0459570000
6	1.0252530000	3.3892090000	0.0458990000
6	−1.0252530000	−3.3892090000	0.0458990000
6	3.4596040000	−0.3380560000	0.7403620000
6	−3.4596040000	0.3380560000	0.7403620000
6	0.0664940000	0.6905380000	3.5078540000
6	−0.0664940000	−0.6905380000	3.5078540000
6	1.3049360000	1.2991710000	3.0654820000
6	−1.3049360000	−1.2991710000	3.0654820000
6	2.3577440000	0.4985790000	2.6383480000
6	−2.3577440000	−0.4985790000	2.6383480000
6	3.1237830000	0.8750210000	1.4650090000
6	−3.1237830000	−0.8750210000	1.4650090000
6	3.4595650000	−0.3382140000	−0.6485190000
6	−3.4595650000	0.3382140000	−0.6485190000
6	0.0666640000	0.6905960000	−3.4160900000
6	−0.0666640000	−0.6905960000	−3.4160900000
6	1.0318930000	−1.5267410000	−2.9717100000
6	−1.0318930000	1.5267410000	−2.9717100000
6	1.0322320000	−1.5262150000	3.0633480000
6	−1.0322320000	1.5262150000	3.0633480000
6	1.3052060000	1.2986420000	−2.9735210000
6	−1.3052060000	−1.2986420000	−2.9735210000
6	2.2187650000	−0.9464440000	−2.5461750000
6	−2.2187650000	0.9464440000	−2.5461750000
6	2.2189240000	−0.9458950000	2.6378970000
6	−2.2189240000	0.9458950000	2.6378970000
6	2.3578230000	0.4980290000	−2.5464820000
6	−2.3578230000	−0.4980290000	−2.5464820000
6	2.9004790000	−1.4647810000	−1.3748680000
6	−2.9004790000	1.4647810000	−1.3748680000
6	2.9006090000	−1.4645510000	1.4667270000
6	−2.9006090000	1.4645510000	1.4667270000
6	3.1237870000	0.8747610000	−1.3731720000
6	−3.1237870000	−0.8747610000	−1.3731720000
6	2.3654280000	−2.5425350000	−0.6800040000
6	−2.3654280000	2.5425350000	−0.6800040000
6	2.3655580000	−2.5424400000	0.7719020000
6	−2.3655580000	2.5424400000	0.7719020000
6	2.8040760000	2.0355910000	−0.6794590000
6	−2.8040760000	−2.0355910000	−0.6794590000
6	2.8040480000	2.0357410000	0.7712820000

Continued on next page

6	−2.8040480000	−2.0357410000	0.7712820000
6	0.9721580000	2.5128380000	−2.2505780000
6	−0.9721580000	−2.5128380000	−2.2505780000
6	0.4741180000	−2.6543600000	2.3425240000
6	−0.4741180000	2.6543600000	2.3425240000
6	0.9718410000	2.5130760000	2.3423950000
6	−0.9718410000	−2.5130760000	2.3423950000
6	0.4737470000	−2.6545830000	−2.2506930000
6	−0.4737470000	2.6545830000	−2.2506930000
6	1.1250980000	−3.1502550000	−1.1282900000
6	−1.1250980000	3.1502550000	−1.1282900000
6	1.7051130000	2.8724710000	1.2193260000
6	−1.7051130000	−2.8724710000	1.2193260000
6	1.1252910000	−3.1501890000	1.2201580000
6	−1.1252910000	3.1501890000	1.2201580000
6	1.7052230000	2.8723450000	−1.1274920000
6	−1.7052230000	−2.8723450000	−1.1274920000
1	8.3455020000	−6.3448040000	1.2093210000
1	−8.3455020000	6.3448040000	1.2093210000
6	5.2008399999	−3.8073610000	4.0094670000
6	−5.2008399999	3.8073610000	4.0094670000
1	6.6936590000	−2.2676700000	4.2839830000
1	−6.6936590000	2.2676700000	4.2839830000
16	7.5014510000	−4.5766310000	2.6666100000
16	−7.5014510000	4.5766310000	2.6666100000
6	4.7724030000	−1.9098320000	5.2943710000
6	−4.7724030000	1.9098320000	5.2943710000
6	3.5577040000	−2.5435590000	5.4233790000
6	−3.5577040000	2.5435590000	5.4233790000
6	5.6952190000	−2.6201660000	4.4997180000
6	−5.6952190000	2.6201660000	4.4997180000
1	4.9839940000	−0.9531500000	5.7501260000
1	−4.9839940000	0.9531500000	5.7501260000
16	2.1090030000	−0.3559450000	6.2490170000
16	−2.1090030000	0.3559450000	6.2490170000
6	5.3269660000	−5.9315890000	2.6236030000
6	−5.3269660000	5.9315890000	2.6236030000
6	5.8464960000	−4.7765130000	3.1615710000
6	−5.8464960000	4.7765130000	3.1615710000
6	7.4691720000	−6.0302970000	1.7505680000
6	−7.4691720000	6.0302970000	1.7505680000
6	6.2531110000	−6.6446430000	1.8247550000
6	−6.2531110000	6.6446430000	1.8247550000
1	6.0225280000	−7.5706760000	1.3200980000
1	−6.0225280000	7.5706760000	1.3200980000

Continued on next page

1	4.3093690000	−6.2525840000	2.7946790000
1	−4.3093690000	6.2525840000	2.7946790000
16	3.5639040000	−4.0449040000	4.5456380000
16	−3.5639040000	4.0449040000	4.5456380000
6	0.1173830000	−1.9096330000	6.6919830000
6	−0.1173830000	1.9096330000	6.6919830000
6	0.4388830000	−0.5697970000	6.6839960000
6	−0.4388830000	0.5697970000	6.6839960000
6	2.3449710000	−2.0608950000	6.0237270000
6	−2.3449710000	2.0608950000	6.0237270000
6	1.1885910000	−2.7480680000	6.3211020000
6	−1.1885910000	2.7480680000	6.3211020000
1	0.8775480000	2.2703780000	6.9105690000
1	−0.8775480000	−2.2703780000	6.9105690000
1	1.1086900000	−3.8221150000	6.2347700000
1	−1.1086900000	3.8221150000	6.2347700000
1	8.3715050000	−6.7984350000	−1.6107400000
1	−8.3715050000	6.7984350000	−1.6107400000
6	5.1744860000	−4.0044000000	−4.0907500000
6	−5.1744860000	4.0044000000	−4.0907500000
1	6.6941760000	−2.4869320000	−4.3410820000
1	−6.6941760000	2.4869320000	−4.3410820000
16	7.5081330000	−4.9104130000	−2.8967520000
16	−7.5081330000	4.9104130000	−2.8967520000
6	4.7504150000	−2.0371450000	−5.2686180000
6	−4.7504150000	2.0371450000	−5.2686180000
6	3.5187070000	−2.6386010000	−5.3910910000
6	−3.5187070000	2.6386010000	−5.3910910000
6	5.6798810000	−2.8042570000	−4.5365370000
6	−5.6798810000	2.8042570000	−4.5365370000
1	4.9725810000	−1.0679650000	−5.6917560000
1	−4.9725810000	1.0679650000	−5.6917560000
16	2.1023720000	−0.4029330000	−6.1586000000
16	−2.1023720000	0.4029330000	−6.1586000000
6	5.3032220000	−6.2147620000	−2.8469630000
6	−5.3032220000	6.2147620000	−2.8469630000
6	5.8285910000	−5.0378270000	−3.3291730000
6	−5.8285910000	5.0378270000	−3.3291730000
6	7.4794170000	−6.4246910000	−2.0845470000
6	−7.4794170000	6.4246910000	−2.0845470000
6	6.2454520000	−7.0039600000	−2.1442070000
6	−6.2454520000	7.0039600000	−2.1442070000
1	6.0143200000	−7.9596140000	−1.6980530000
1	−6.0143200000	7.9596140000	−1.6980530000
1	4.2719200000	−6.5013070000	−2.9956250000

Continued on next page

1	−4.2719200000	6.5013070000	−2.9956250000
16	3.5140700000	−4.1756540000	−4.5774940000
16	−3.5140700000	4.1756540000	−4.5774940000
6	0.0762850000	−1.9112480000	−6.6034380000
6	−0.0762850000	1.9112480000	−6.6034380000
6	0.4268070000	−0.5788510000	−6.5918690000
6	−0.4268070000	0.5788510000	−6.5918690000
6	2.3052870000	−2.1155410000	−5.9553960000
6	−2.3052870000	2.1155410000	−5.9553960000
6	1.1316310000	−2.7749630000	−6.2471360000
6	−1.1316310000	2.7749630000	−6.2471360000
1	0.9264830000	2.2494830000	−6.8221920000
1	−0.9264830000	−2.2494830000	−6.8221920000
1	1.0309520000	−3.8482650000	−6.1743800000
1	−1.0309520000	3.8482650000	−6.1743800000

Table S9: Cartesian Coordinates for T10-C_{60}-T10 Complex with $C_2(y)$ Symmetry in Å

6	3.5036820000	0.0138740000	−0.5360570000
6	−3.5036820000	0.0138740000	0.5360570000
6	3.0281490000	0.0136730000	−1.8433610000
6	−3.0281490000	0.0136730000	1.8433610000
6	1.1883110000	0.7082950000	3.2688000000
6	−1.1883110000	0.7082950000	−3.2688000000
6	0.6509490000	3.4656770000	−0.2373170000
6	−0.6509490000	3.4656770000	0.2373170000
6	0.9322700000	3.0265250000	−1.5893050000
6	−0.9322700000	3.0265250000	1.5893050000
6	0.1036430000	2.6061290000	2.4134620000
6	−0.1036430000	2.6061290000	−2.4134620000
6	0.0696190000	1.4342960000	−3.2499650000
6	−0.0696190000	1.4342960000	3.2499650000
6	1.1888900000	−0.6807820000	3.2686290000
6	−1.1888900000	−0.6807820000	−3.2686290000
6	0.6511090000	−3.4382620000	−0.2369460000
6	−0.6511090000	−3.4382620000	0.2369460000
6	1.7360880000	−3.0006110000	0.6178340000
6	−1.7360880000	−3.0006110000	−0.6178340000
6	1.7343280000	3.0275960000	0.6190630000
6	−1.7343280000	3.0275960000	−0.6190630000
6	0.9299110000	−2.9984480000	−1.5891610000
6	−0.9299110000	−2.9984480000	1.5891610000
6	1.4700470000	−2.5779350000	1.9147700000
6	−1.4700470000	−2.5779350000	−1.9147700000
6	1.4681480000	2.6052350000	1.9155650000
6	−1.4681480000	2.6052350000	−1.9155650000
6	0.1057160000	−2.5783980000	2.4129670000
6	−0.1057160000	−2.5783980000	−2.4129670000
6	2.1397500000	−1.4056930000	2.4432310000
6	−2.1397500000	−1.4056930000	−2.4432310000
6	2.1388030000	1.4332410000	2.4435140000
6	−2.1388030000	1.4332410000	−2.4435140000
6	0.0686580000	−1.4068170000	−3.2496630000
6	−0.0686580000	−1.4068170000	3.2496630000
6	3.0500060000	−0.7114300000	1.6533910000
6	−3.0500060000	−0.7114300000	−1.6533910000
6	3.0495130000	0.7390650000	1.6536390000
6	−3.0495130000	0.7390650000	−1.6536390000
6	1.2734200000	−0.7116560000	−3.2303170000
6	−1.2734200000	−0.7116560000	3.2303170000
6	1.2740100000	0.7391040000	−3.2304300000

Continued on next page

6	−1.2740100000	0.7391040000	3.2304300000
6	2.1897900000	−2.2811990000	−1.5716280000
6	−2.1897900000	−2.2811990000	1.5716280000
6	2.6867910000	2.3084920000	−0.2051120000
6	−2.6867910000	2.3084920000	0.2051120000
6	2.1911350000	2.3087890000	−1.5711310000
6	−2.1911350000	2.3087890000	1.5711310000
6	2.6873170000	−2.2809570000	−0.2062550000
6	−2.6873170000	−2.2809570000	0.2062550000
6	3.3310520000	−1.1590490000	0.3009100000
6	−3.3310520000	−1.1590490000	−0.3009100000
6	2.3579710000	1.1867820000	−2.3735020000
6	−2.3579710000	1.1867820000	2.3735020000
6	3.3306840000	1.1866520000	0.3013980000
6	−3.3306840000	1.1866520000	−0.3013980000
6	2.3572720000	−1.1592700000	−2.3736030000
6	−2.3572720000	−1.1592700000	2.3736030000
16	0.6129100000	2.3218760000	12.9084950000
16	−0.6129100000	2.3218760000	−12.9084950000
6	0.8604170000	1.6875190000	17.4922200000
6	−0.8604170000	1.6875190000	−17.4922200000
6	0.4095850000	1.7981470000	−17.9786199999
6	−0.4095850000	1.7981470000	17.9786199999
6	0.3168240000	1.9377320000	−15.4921130000
6	−0.3168240000	1.9377320000	15.4921130000
6	0.9159870000	1.7661190000	16.0791340000
6	−0.9159870000	1.7661190000	−16.0791340000
1	1.8319140000	1.6979790000	15.5094490000
1	−1.8319140000	1.6979790000	−15.5094490000
1	1.7262380000	1.5378120000	18.1208029999
1	−1.7262380000	1.5378120000	−18.1208029999
16	0.9083340000	4.9266410000	5.5643910000
16	−0.9083340000	4.9266410000	−5.5643910000
6	1.1711600000	3.0850040000	9.8124579999
6	−1.1711600000	3.0850040000	−9.8124579999
6	0.1078980000	2.9943480000	−10.3174270000
6	−0.1078980000	2.9943480000	10.3174270000
6	0.0091000000	3.8997670000	−7.9725030000
6	−0.0091000000	3.8997670000	7.9725030000
6	1.2265590000	3.5916990000	8.4991380000
6	−1.2265590000	3.5916990000	−8.4991380000
1	2.1487810000	3.7420120000	7.9556780000
1	−2.1487810000	3.7420120000	−7.9556780000
1	2.0465020000	2.8088070000	10.3832770000
1	−2.0465020000	2.8088070000	−10.3832770000

Continued on next page

6	1.4233490000	6.1426840000	2.6149020000
6	−1.4233490000	6.1426840000	−2.6149020000
6	0.1540480000	6.0297120000	3.1386350000
6	−0.1540480000	6.0297120000	−3.1386350000
6	0.1842500000	6.6135680000	0.6966030000
6	−0.1842500000	6.6135680000	−0.6966030000
6	1.4405720000	6.4694100000	1.2450280000
6	−1.4405720000	6.4694100000	−1.2450280000
1	2.3458800000	6.5160720000	0.6567330000
1	−2.3458800000	6.5160720000	−0.6567330000
1	2.3151260000	5.9388630000	3.1904580000
1	−2.3151260000	5.9388630000	−3.1904580000
16	1.5537420000	2.0057280000	−16.7134089999
16	−1.5537420000	2.0057280000	16.7134089999
6	1.8772860000	2.2638770000	−13.5136369999
6	−1.8772860000	2.2638770000	13.5136369999
6	0.6402820000	2.1255710000	−14.1003209999
6	−0.6402820000	2.1255710000	14.1003209999
6	1.8216750000	2.5433140000	−12.1338690000
6	−1.8216750000	2.5433140000	12.1338690000
1	2.8006500000	2.1834070000	−14.0697470000
1	−2.8006500000	2.1834070000	14.0697470000
16	1.2596780000	3.5362770000	−9.1290900000
16	−1.2596780000	3.5362770000	9.1290900000
6	1.5772850000	4.7998030000	−6.1942980000
6	−1.5772850000	4.7998030000	6.1942980000
6	0.3356920000	4.4797910000	−6.7002109999
6	−0.3356920000	4.4797910000	6.7002109999
6	0.5396610000	2.6227340000	−11.6369340000
6	−0.5396610000	2.6227340000	11.6369340000
6	1.5326900000	5.3916330000	−4.9186160000
6	−1.5326900000	5.3916330000	4.9186160000
1	2.6980350000	2.7016380000	−11.5211869999
1	−2.6980350000	2.7016380000	11.5211869999
1	2.4950310000	4.6127750000	−6.7336860000
1	−2.4950310000	4.6127750000	6.7336860000
16	1.0351160000	6.4029240000	−1.9225270000
16	−1.0351160000	6.4029240000	1.9225270000
6	0.2556820000	5.5436410000	−4.4241390000
6	−0.2556820000	5.5436410000	4.4241390000
1	2.4133090000	5.6926440000	−4.3688530000
1	−2.4133090000	5.6926440000	4.3688530000
1	0.7401330000	1.7507640000	−19.0039319999
1	−0.7401330000	1.7507640000	19.0039319999
16	1.3272000000	−2.4151610000	12.8628269999

Continued on next page

16	−1.3272000000	−2.4151610000	−12.8628269999
6	1.9386410000	−1.8698090000	17.4177870000
6	−1.9386410000	−1.8698090000	−17.4177870000
6	0.7016520000	−1.8910730000	17.9933009999
6	−0.7016520000	−1.8910730000	−17.9933009999
6	0.6060330000	−2.0072110000	15.5057480000
6	−0.6060330000	−2.0072110000	−15.5057480000
6	1.8870150000	−1.9339880000	16.0038090000
6	−1.8870150000	−1.9339880000	−16.0038090000
1	2.7625700000	−1.9287770000	15.3700399999
1	−2.7625700000	−1.9287770000	−15.3700399999
1	2.8559540000	−1.7941800000	17.9837540000
1	−2.8559540000	−1.7941800000	−17.9837540000
16	1.0910440000	−4.9315260000	5.5250690000
16	−1.0910440000	−4.9315260000	−5.5250690000
6	1.6429110000	−3.1538150000	9.7442970000
6	−1.6429110000	−3.1538150000	−9.7442970000
6	0.4013660000	−3.0100910000	10.3240510000
6	−0.4013660000	−3.0100910000	−10.3240510000
6	0.3225430000	−3.9009910000	7.9727410000
6	−0.3225430000	−3.9009910000	−7.9727410000
6	1.5986590000	−3.6494120000	8.4273960000
6	−1.5986590000	−3.6494120000	−8.4273960000
1	2.4801330000	−3.8358080000	7.8301600000
1	−2.4801330000	−3.8358080000	−7.8301600000
1	2.5617190000	−2.9215680000	10.2641069999
1	−2.5617190000	−2.9215680000	−10.2641069999
6	1.4806510000	−6.1223710000	2.5829580000
6	−1.4806510000	−6.1223710000	−2.5829580000
6	0.2246070000	−6.0037470000	3.1351610000
6	−0.2246070000	−6.0037470000	−3.1351610000
6	0.1982470000	−6.5859010000	0.6925910000
6	−0.1982470000	−6.5859010000	−0.6925910000
6	1.4659460000	−6.4451470000	1.2138160000
6	−1.4659460000	−6.4451470000	−1.2138160000
1	2.3579970000	−6.4942820000	0.6057800000
1	−2.3579970000	−6.4942820000	−0.6057800000
1	2.3860450000	−5.9232500000	3.1385300000
1	−2.3860450000	−5.9232500000	−3.1385300000
16	0.5419590000	−1.9974860000	−16.8123259999
16	−0.5419590000	−1.9974860000	16.8123259999
6	1.1070830000	−2.2088160000	−13.6372690000
6	−1.1070830000	−2.2088160000	13.6372690000
6	0.1733390000	−2.1559500000	14.1390630000
6	−0.1733390000	−2.1559500000	−14.1390630000

Continued on next page

6	1.1632010000	−2.4780420000	−12.2549440000
6	−1.1632010000	−2.4780420000	12.2549440000
1	1.9835580000	−2.0768200000	−14.2558640000
1	−1.9835580000	−2.0768200000	14.2558640000
16	0.8421730000	−3.4914120000	−9.2024300000
16	−0.8421730000	−3.4914120000	9.2024300000
6	1.3598200000	−4.7464230000	−6.2635650000
6	−1.3598200000	−4.7464230000	6.2635650000
6	0.0889900000	−4.4612140000	−6.7162260000
6	−0.0889900000	−4.4612140000	6.7162260000
6	0.0749550000	−2.6294370000	11.6709110000
6	−0.0749550000	−2.6294370000	−11.6709110000
6	1.3855650000	−5.3351650000	−4.9844410000
6	−1.3855650000	−5.3351650000	4.9844410000
1	2.0872720000	−2.5747850000	−11.7025500000
1	−2.0872720000	−2.5747850000	11.7025500000
1	2.2480520000	−4.5399390000	−6.8437420000
1	−2.2480520000	−4.5399390000	6.8437420000
16	0.9949580000	−6.3716680000	−1.9453910000
16	−0.9949580000	−6.3716680000	1.9453910000
6	0.1346920000	−5.5150180000	−4.4346130000
6	−0.1346920000	−5.5150180000	4.4346130000
1	2.2962070000	−5.6126300000	−4.4724910000
1	−2.2962070000	−5.6126300000	4.4724910000
1	0.4493610000	−1.8320030000	19.0399870000
1	−0.4493610000	−1.8320030000	−19.0399870000

References

(1) Weigend, F.; Ahlrichs, R. *Phys. Chem. Chem. Phys.* **2005**, *7*, 3297.

(2) Francl, M. M.; Pietro, W. J.; Hehre, W. J.; Binkley, S. J.; Gordon, M. S.; DeFrees, D. J.; Pople, J. A. *J. Chem. Phys.* **1982**, *77*, 3654.

(3) McLean, A.; Chandler, G. *J. Chem. Phys.* **1980**, *72*, 5639.
